# Crystal structures of glycoprotein D of equine alphaherpesviruses reveal potential binding sites to the entry receptor MHC-I

**DOI:** 10.3389/fmicb.2023.1197120

**Published:** 2023-05-11

**Authors:** Viviane Kremling, Bernhard Loll, Szymon Pach, Ismail Dahmani, Christoph Weise, Gerhard Wolber, Salvatore Chiantia, Markus C. Wahl, Nikolaus Osterrieder, Walid Azab

**Affiliations:** ^1^Institut für Virologie, Robert von Ostertag-Haus, Zentrum für Infektionsmedizin, Freie Universität Berlin, Berlin, Germany; ^2^Laboratory of Structural Biochemistry, Freie Universität Berlin, Berlin, Germany; ^3^Institute of Pharmacy (Pharmaceutical Chemistry), Freie Universität Berlin, Berlin, Germany; ^4^Universität Potsdam, Institut für Biochemie und Biologie, Potsdam, Brandenburg, Germany; ^5^BioSupraMol Core Facility, Bio-Mass Spectrometry, Freie Universität Berlin, Berlin, Germany; ^6^Helmholtz-Zentrum Berlin für Materialien und Energie, Macromolecular Crystallography, Berlin, Germany; ^7^Department of Infectious Diseases and Public Health, City University of Hong Kong, Kowloon, Hong Kong SAR, China

**Keywords:** host-pathogen interaction, virus entry, glycoprotein D, alphaherpesviruses, receptor binding

## Abstract

Cell entry of most alphaherpesviruses is mediated by the binding of glycoprotein D (gD) to different cell surface receptors. Equine herpesvirus type 1 (EHV-1) and EHV-4 gDs interact with equine major histocompatibility complex I (MHC-I) to initiate entry into equine cells. We have characterized the gD-MHC-I interaction by solving the crystal structures of EHV-1 and EHV-4 gDs (gD1, gD4), performing protein–protein docking simulations, surface plasmon resonance (SPR) analysis, and biological assays. The structures of gD1 and gD4 revealed the existence of a common V-set immunoglobulin-like (IgV-like) core comparable to those of other gD homologs. Molecular modeling yielded plausible binding hypotheses and identified key residues (F213 and D261) that are important for virus binding. Altering the key residues resulted in impaired virus growth in cells, which highlights the important role of these residues in the gD-MHC-I interaction. Taken together, our results add to our understanding of the initial herpesvirus-cell interactions and will contribute to the targeted design of antiviral drugs and vaccine development.

## 1. Introduction

An essential step for virus replication is the entry process into host cells. In herpesviruses, more specifically alphaherpesviruses, cell entry is a complex multistep process that requires a stepwise contribution of five out of 12 envelope glycoproteins, namely glycoprotein B (gB), gC, gD, and the heterodimer gH/gL (Osterrieder and Van de Walle, 2010). Of these, gD is the (main) receptor-binding protein that interacts with the cell receptors and triggers the subsequent fusion process with cell membrane and/or uptake by endocytosis (Cole and Grose, 2003; Frampton et al., 2007; Azab et al., 2015).

Equine herpesvirus type 1 (EHV-1) and EHV-4 use equine major histocompatibility complex class I (MHC-I) as an entry receptor, however, no details of the molecular binding mode are available (Kurtz et al., 2010; Sasaki et al., 2011; Azab et al., 2014). Only few other viruses are known to utilize MHC molecules as binding receptors but not as entry receptors. Coxsackievirus A9 requires MHC-I and GRP78 as co-receptors for virus internalization (Triantafilou et al., 2002), Simian virus 40 (SV40) binds to cellular MHC-I, however, MHC-I does not mediate virus entry (Atwood and Norkin, 1989; Norkin, 1999). The fiber knob of Adenovirus type 5 (AdV-5) binds to the α2 region of human leukocyte antigen (HLA; Hong et al., 1997), and the functional gD homolog gp42 in Epstein–Barr Virus (EBV) binds to MHC-II to activate membrane fusion (Mullen et al., 2002).

MHC-I seems to be an unlikely receptor for viral entry since it is present on all somatic cells (David-Watine et al., 1990) and therefore does not confer tissue specificity. Additionally, it is one of the most polymorphic mammalian proteins with 10 to 25% difference in the amino acid sequence (Gilcrease, 2007; Tallmadge et al., 2010). Typically, MHC-I plays a crucial role in the adaptive immunity by presenting proteolytically processed intracellular proteins on the cell surface to T-cells and natural killer cells (Bjorkman and Parham, 1990). In case of an infected cell, virus-derived peptides are presented and the recognition by T-cell receptor (TCR) initiates an immune response (Germain and Margulies, 1993). Although utilized by EHV-1 and EHV-4 as entry receptors, not all MHC-I genes support the entry of both viruses (Kurtz et al., 2010; Sasaki et al., 2011; Azab et al., 2014). Interestingly, the residue A173 in the α2 region of MHC-I seems to be necessary but not enough to trigger virus entry (Ellis et al., 1995; Sasaki et al., 2011; Azab et al., 2014).

EHV-1 and EHV-4 are important pathogens that cause great suffering in *Equidae* and other mammals and result in huge economic losses to the equine industry (Patel and Heldens, 2005). Efforts have been made to find efficient vaccines against both viruses (Kydd et al., 2006). However, the protection is usually limited in time and efficacy; frequent outbreaks occur also in vaccinated horses (Burrows and Goodridge, 1974; Allen, 1986; Goehring et al., 2010; Goodman et al., 2012).

Here, we present crystal structures of free gD1 and gD4, which show a similar fold as other gD proteins from related viruses such as herpes simplex virus type 1 [HSV-1; PDB-ID 2C36, (Krummenacher et al., 2005)], pseudorabies virus [PrV, PDB-ID 5X5V, (Yue et al., 2020)], and bovine herpesvirus type 1 [BoHV-1, PDB-ID 6LS9, (Yue et al., 2020)]. We further measured dissociation constants (in a micromolar range) for recombinant gD1/gD4 and C-terminally truncated gD4 binding to equine MHC-I by surface plasmon resonance spectroscopy (SPR). No increased binding affinity was observed for the truncated protein as was the case for gD of HSV-1, HSV-2, and PrV (Lu et al., 2014; Li et al., 2017), suggesting a structurally different mode of binding during entry into host cells. Cell culture assays showed that recombinant gD1 and gD4 as well as truncated gD4 can inhibit viral replication *in vitro*, where again the truncated version did not perform better than the full-length protein. The crystal structures were further used for *in silico* docking analyses to equine MHC-I. Based on these docking positions, viral mutants with point mutations at position F213 or D261 were produced and displayed significant growth impairments which support the proposed mode of binding of gD1 and gD4 to MHC-I.

## 2. Results

### 2.1. Crystal structure of unbound EHV-1 and EHV-4 gD

**Figure 1:**
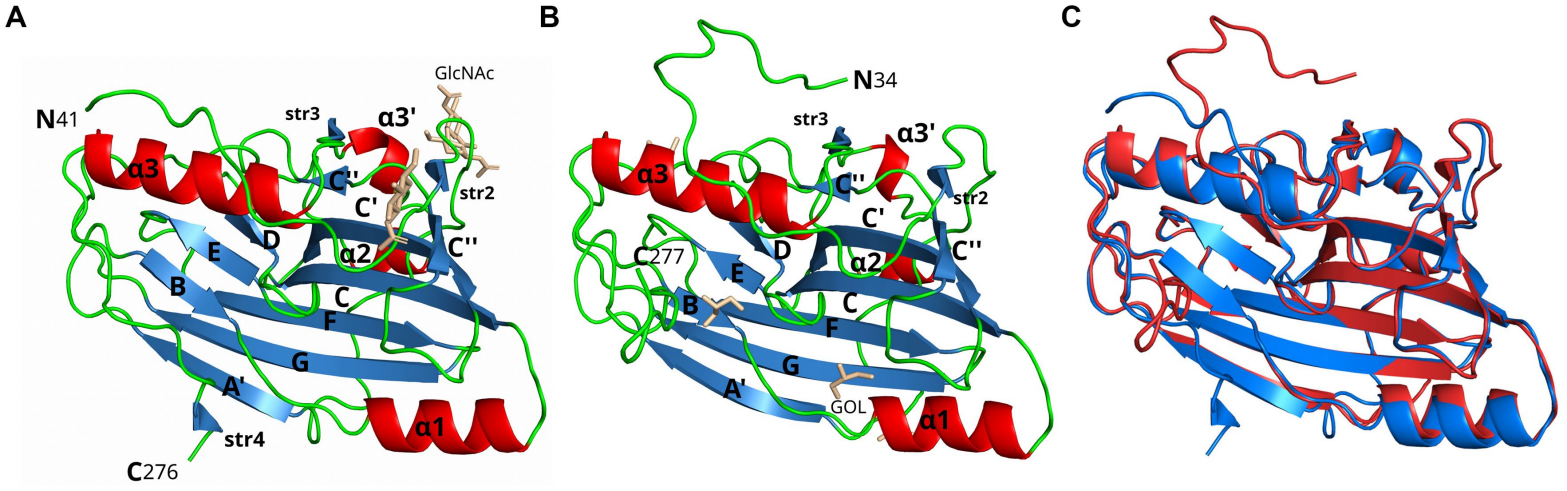
Crystal structure comparison of gD1 and gD4. Cartoon representation of **(A)** gD1 (2.25 Å resolution, PDB-ID: 6SQJ, chain B) and **(B)** gD4 (1.9 Å resolution, PDB-ID: 6TM8) crystal structures. Molecule orientation is identical and secondary structures were assigned with dssp (Kabsch and Sander, 1983). Helices are displayed in red, sheets in blue, and loops in green. N-acetylglucosamine (GlcNAc) and glycerol (GOL) molecules are shown in stick representation in beige. **(C)** Superposition of the crystal structures of gD1 (blue, PDB-ID 6SQJ) and gD4 (red, PDB-ID 6TM8). GlcNAc and glycerol molecules are not shown.

Recombinant gD1 and gD4 lacking the transmembrane region were produced in insect cells, purified by affinity and size exclusion chromatography and used for crystallization experiments (Figure 1). To evaluate the correct identity, sequence, and molecular mass of gD1, gD4, and equine MHC-I, mass spectrometry (MS) analysis was conducted (Supplementary Figure S1; Table S1). Recombinant equine MHC-I 3.1 (Eqca-1*00101) including a peptide (SDYVKVSNI) linked to the β2-microglobulin (β2m) region was produced in insect cells as well and purified in the same manner as gD1 and gD4.

A 2.25 Å resolution diffraction data set was collected for a gD1 crystal, and the gD1 structure (Figure 1A) was determined using the structure coordinates of HSV-1 gD (PDB-ID 2C36) for molecular replacement and refined to an R_work_ of 20.3% and R_free_ of 25.7% (Table 1, PDB-ID 6SQJ). The crystal structure of gD1 contains two gD molecules per asymmetric unit. In solution only the monomeric form was observed by size exclusion chromatography (SEC)-multi-angle static light scattering (MALS; Supplementary Figure S6). In the crystal structure, two ions interpreted as magnesium are trapped between the molecules forming the dimer which are coordinated by residues E242 and D261 of both protein chains together with water molecules. In chain A, the terminal residues E31 to R38, P281 to T348, and the loop region A N71 to N76 could not be modeled due to a lack of electron density. The same is true for the termini of chain B E31 to R40 and N277 to T348 (Supplementary Figure S7). N-linked glycosylations are visible at the predicted sites N53 and N61 (Flowers et al., 1991) which are conserved between gD1 and gD4 (Supplementary Figure S7) but not in gDs of other alphaherpesviruses (Supplementary Figure S8).

**Table 1:** Crystallographic data collection and model refinement statistics.

PDB-ID	gD1 6SQJ	gD4 6TM8
**Data collection**		
Wavelength [Å]	1.0332	0.91841
Temperature [K]	100	100
Space group	P2_1_2_1_2_1_	P2_1_2_1_2_1_
Unit cell parameters		
a, b, c [Å]	71.9; 94.5; 101.3	73.1; 59.6; 69.7
α = β = γ [°]	90	90
Resolution range [Å]	50.00–2.24 (2.38–2.24)^a^	50.00–1.90 (2.01–1.90)
Reflections^a^	218,509 (33,751)	138,685 (10,835)
Unique reflections^a^	33,402 (5,140)	23,671 (1,810)
Completeness [%]^a^	99.1 (95.8)	95.6 (78.2)
Multiplicit^a^	6.5 (6.6)	5.9 (3.5)
**Data quality**^a^		
I/σ(I)^a^	11.71 (0.92)	8.96 (0.96)
Rmeas [%]^a^	13.5 (199)	17.4 (126.8)
CC_1/2_^a^	99.8 (58.6)	99.5 (40.9)
Wilson B factor [Å^2^]	53.3	32
**Refinement gD1 gD4**		
Resolution range [Å]^a^	50.00–2.24 (2.33–2.24)	50.00–1.90 (2.01–1.90)
Reflections^a^	33,399 (3,181)	23,642 (1,792)
Test set (5%)^a^	1,669 (159)	1,182 (89)
R_work_ [%]^a^	20.3 (33.8)	17.5 (30.0)
R_free_ [%]^a^	25.7 (34.0)	21.5 (37.2)
**Contents of asymmetric unit**		
Molecules, residues, atoms	2; 477; 4,049	1; 244; 2,037
Mg^2+^, GlcNAc molecules, glycerol	2; 5; −	-; −; 4
Water molecules	132	174
**Mean Temperature factors [Å**^2^**]**^b^		
All atoms	58.7	31.1
Macromolecules	58	30.4
Ligands	106.7	49.9
Water oxygens	53.5	36.0
RMSD from target geometry		
Bond length [Å]	0.007	0.012
Bond angles [°]	0.84	1.04
**Validation statistics**^c^		
**Ramachandran plot**		
Residues in allowed regions [%]	2.8	2.5
Residues in favored regions [%]	97.2	97.5
MOLPROBITIY clashscore^d^	3.23	3.9

^a^Data for the highest resolution shell in parenthesis.^b^Calculated with PHENIX (Adams et al., 2010).^c^Calculated with MOLPROBITY (Chen et al., 2010).^d^Clash score is the number of serious steric overlaps (> 0.4) per 1,000 atoms.

Glycoprotein D4 was crystallized with one protein per asymmetric unit. The structure was determined at a resolution of 1.9 Å (Table 1, PDB-ID 6TM8, Figure 1B) using the coordinates of gD1 structure for molecular replacement and refined to an R_work_ of 17.5% and R_free_ of 21.5% (Table 1). In total 244 residues could be modeled (R34 to R277, Supplementary Figure S7).

In the structures of gD1 and gD4, six cysteines were found to form three disulfide bonds at sites conserved in members of the gD plolypeptide family: C87/C209, C126/C223, and C138/C147 (Carfi et al., 2001; Li et al., 2017; Yue et al., 2020; Supplementary Figure S7). The overall folds of gD1 and gD4 are very similar with a root-mean-square deviation (rmsd) of 0.7 Å for 220 common Cα atoms (Figure 1C). The cores consist of a nine-stranded (A′, B, C, C′, C″, D, E, F, and G) β-barrel, arranged in a typical V-like Ig fold, flanked by N- and C-terminal extensions with loops, α-helices (α1, α2, α3’, and α3), and small β-strands (str2-4). The termini in both structures point in opposite directions (Figure 1C) and the unresolved C-termini are predicted to be unstructured by Foldindex (Prilusky et al., 2005).

### 2.2. Comparison of gD1, gD4, and homolog structures

**Figure 2:**
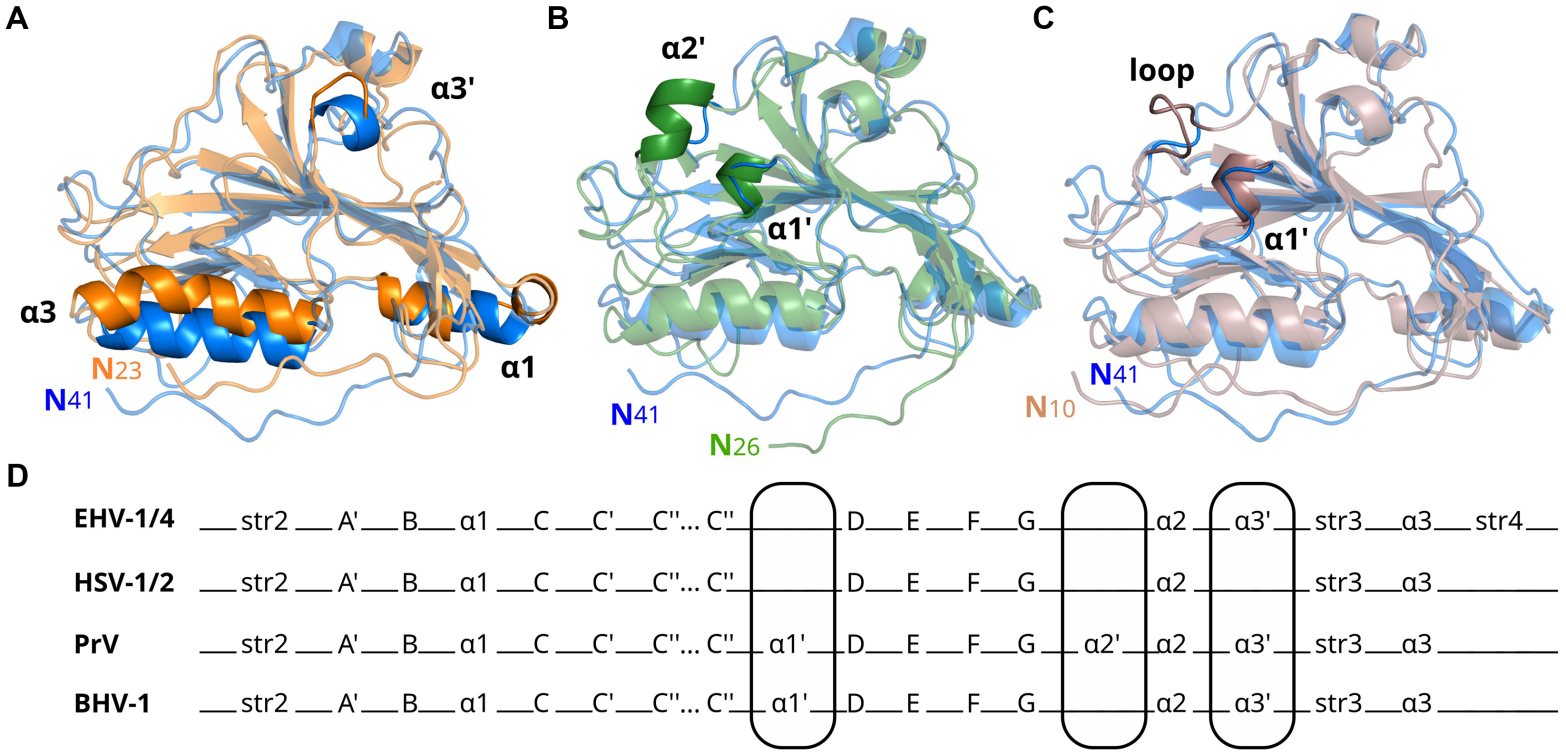
Glycoprotein D from alphaherpesviruses have similar secondary structures. Shown are superpositions of crystal structures in cartoon representation of gD from EHV-1 (blue, PDB-ID 6SQJ) with **(A)** HSV-1 (orange, PDB-ID 2C36), **(B)** PrV (green, PDB-ID 5X5V), and **(C)** BoHV-1 (brown, PDB-ID 6LS9) gD. Main differences in global fold are highlighted. **(D)** Comparison of secondary structure elements of EHV-1/4, HSV-1/2, PrV, and BoHV-1 gD. Main differences in global fold are encircled.

The amino acid sequence identity between EHV-1 and EHV-4 gDs is 76%, much higher than compared to HSV-1 (25%, GenBank AAK19597), PrV (34%, GenBank AEM64108) or BoHV-1 (31%, GenBank NP045370; Supplementary Figure S8). While the global folds of gDs of these different viruses are very similar (Figure 2), the number and positions of α-helices differ. Compared to EHV-1 and EHV-4 gDs, there is an extra helix termed α1’ present in PrV and BoHV-1 gDs. PrV gD has an exclusive α2’ helix which cannot be observed in other gD structures elucidated so far. In HSV-1 and HSV-2 gDs, the α3’ helix is missing but is present in EHV-1, EHV-4, PrV, and BoHV-1 gDs. In HSV-1 and HSV-2 gDs, the α1 helix is split and the α3 helix is kinked in HSV-1 (Figure 2D and Supplementary Figure S9) which is not seen in the other known gD structures.

The six disulfide bonds C87/C209, C126/C223, and C138/C147 are conserved across EHV-1, EHV-4, PrV, HSV-1, HSV-2, and BoHV-1 gDs, while the predicted and resolved glycosylation sites in the crystal structure of gD1 and gD4 are only conserved between EHV-1 and EHV-4 (N52, N61, N297, N386; Supplementary Figures S7, S8). Between gD1 and gD4, also the magnesium-coordinating residues seen in the gD1 monomer-monomer interface are conserved.

### 2.3. Soluble gD1 and gD4 engage recombinant MHC-I with relatively weak binding affinities

gD binding affinities of different alphaherpesviruses to their receptors are known to differ greatly. For example, PrV gD binds human nectin-1 in the nanomolar range (Connolly et al., 2001). HSV-1 gD interacts more weakly, in a micromolar range, with nectin-1 and herpesvirus entry mediator (HVEM; Krummenacher et al., 2005) similarly to BoHV-1 gD with nectin-1 (Yue et al., 2020; Table 2). For HSV-1, HSV-2, and PrV gDs, it has been demonstrated that C-terminal truncation of the proteins increases the binding affinities up to 100-fold (Table 2).

**Table 2:** Comparison of dissociation constants ( $K_{d}^{\mathrm{app}}$ ) of alphaherpesviruses gDs binding their respective receptors measured by SPR.

gD origin	Receptor	*K*_d_^app^(nM)	Reference
EHV-1 (349)	MHC-I	7,000 ± 2,000	This study
EHV-4 (349)	MHC-I	5,800 ± 760	This study
HSV-1 (306)	HVEM	3,200 ± 600	Willis et al. (1998)
HSV-1 (306)	HVEM	4,000	Krummenacher et al. (2005)
HSV-1 (306)	Nectin-1	2,700 ± 200	Whitbeck et al. (1999)
HSV-1 (306)	Nectin-1	1,800	Krummenacher et al. (2005)
HSV-2 (306)	HVEM	1,500	Willis et al. (1998)
PrV (354)	Nectin-1	130 ± 70	Connolly et al. (2001)
PrV (337)	Nectin-1	191	Li et al. (2017)
PrV (337)	SW-nectin-1	301	Li et al. (2017)
BoHV-1 (301)	Nectin-1	879 ± 101	Yue et al. (2020)
BoHV-1 (301)	BO-nectin-1	341 ± 106	Yue et al. (2020)
EHV-4 (280)	MHC-I	6,700 ± 750	This study
HSV-1 (285)	HVEM	37	Rux et al. (1998)
HSV-1 (285)	HVEM	110	Krummenacher et al. (2005)
HSV-1 (285)	Nectin-1	38	Krummenacher et al. (1999)
HSV-1 (285)	Nectin-1	70	Krummenacher et al. (2005)
HSV-1 (285)	Nectin-1	17.1	Zhang et al. (2011)
HSV-1 (285)	Nectin-1	12.5	Lu et al. (2014)
HSV-2 (285)	Nectin-1	19.1	Lu et al. (2014)
PrV (284)	Nectin-1	16.1	Li et al. (2017)
PrV (284)	SW-nectin-1	18.4	Li et al. (2017)
BoHV-1 (274)	Nectin-1	701 ± 68	Yue et al. (2020)
BoHV-1 (274)	BO-nectin-1	489 ± 157	Yue et al. (2020)

SW, swine; BO, bovine; MHC-I = equine (Eqca-1*00101), nectin-1/HVEM = human. The C-terminal truncation of proteins is displayed in brackets under’ gD origin’. The upper part of the table represents full-length proteins, the bottom part truncated proteins.

To study the interaction of soluble gD1 and gD4 with recombinant equine MHC-I, a surface plasmon resonance (SPR) binding assay was conducted. α-chains together with β2m with linked peptide of equine MHC-I 3.1 were produced in insect cells and purified by immobilized metal ion affinity chromatography (IMAC) and SEC (Supplementary Figures S3D, S4D). Additionally, the receptor affinity of a C-terminally truncated EHV-4 gD, gD4_36-280,_ was tested. The truncated protein was produced in the same manner as gD1 and gD4 originally with the goal to crystallize it because the flexible C-terminus was suspected to hinder crystallization of gD1 and gD4. However, shortly after the production of the truncated gD4_36-280,_ crystal structures were obtained for both gD1 and gD4 proteins. Therefore, only for gD4_36-280_ receptor binding kinetics were determined. Another truncated version, gD4_45-276_, could not be produced in insect cells, suggesting that the protein failed to fold properly. Binding analyses for soluble gDs were conducted by using a protein dilution series in a range of 0 to approximately 10 μM.

**Figure 3:**
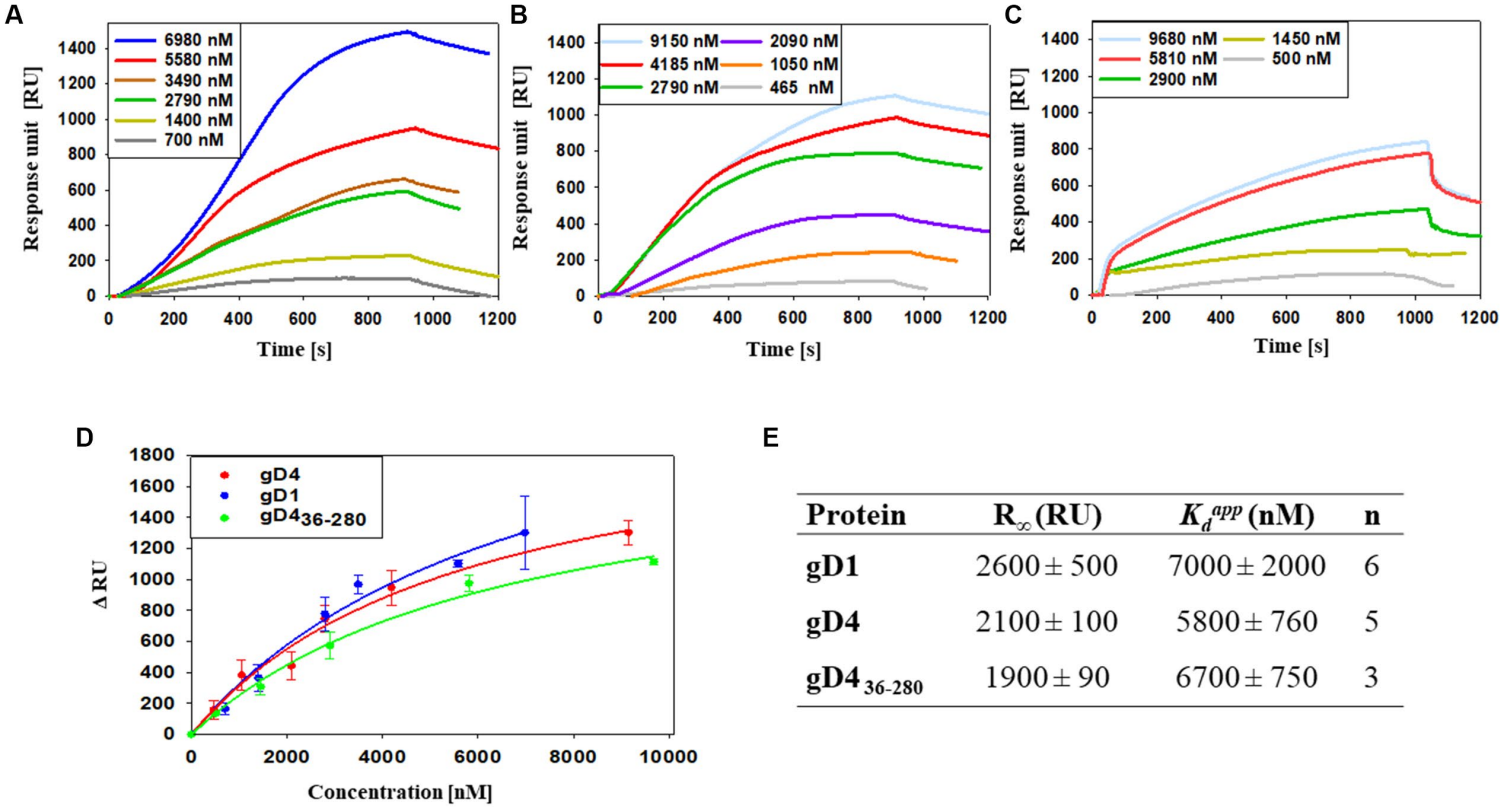
Binding affinities of gD1, gD4 and gD4_36-280_ to the entry receptor MHC-I are in micromolar range. **(A–C)** Representative SPR sensorgram profiles of recombinant gDs binding to amine-coupled recombinant MHC-I. Data were collected for several independent experiments [**(A)** gD1 *n* = 6, **(B)** gD4 *n* = 5, **(C)** gD4_36-280_ *n* = 3]. **(D)** Representative binding curves for different gD concentrations. Displayed are means with standard deviation (SD). The solid lines represent a fit of a 1:1 binding model to the data. **(E)** Parameters obtained from SPR binding curves of gD1, gD4, and gD436-280. R∞ is the maximum signal obtained from the bound protein; $K_{d}^{\mathrm{app}}$ is the apparent equilibrium dissociation constant, n corresponds to the number of independent experiments.

Apparent dissociation constants ( $K_{d}^{\mathrm{app}}$ ) of 7,000 ± 2,000 nM and 5,800 ± 760 nM were calculated for gD1 and gD4, respectively (Figures 3A,B,D,E). The truncated gD4 version, gD4_36-280_, exhibited a receptor binding affinity to MHC-I in the same order of magnitude (6,700 ± 750 nM; Figures 3C–E).

### 2.4. Recombinant gD1, gD4, gD4_36-280_ can block cell surface MHC-I

**Figure 4:**
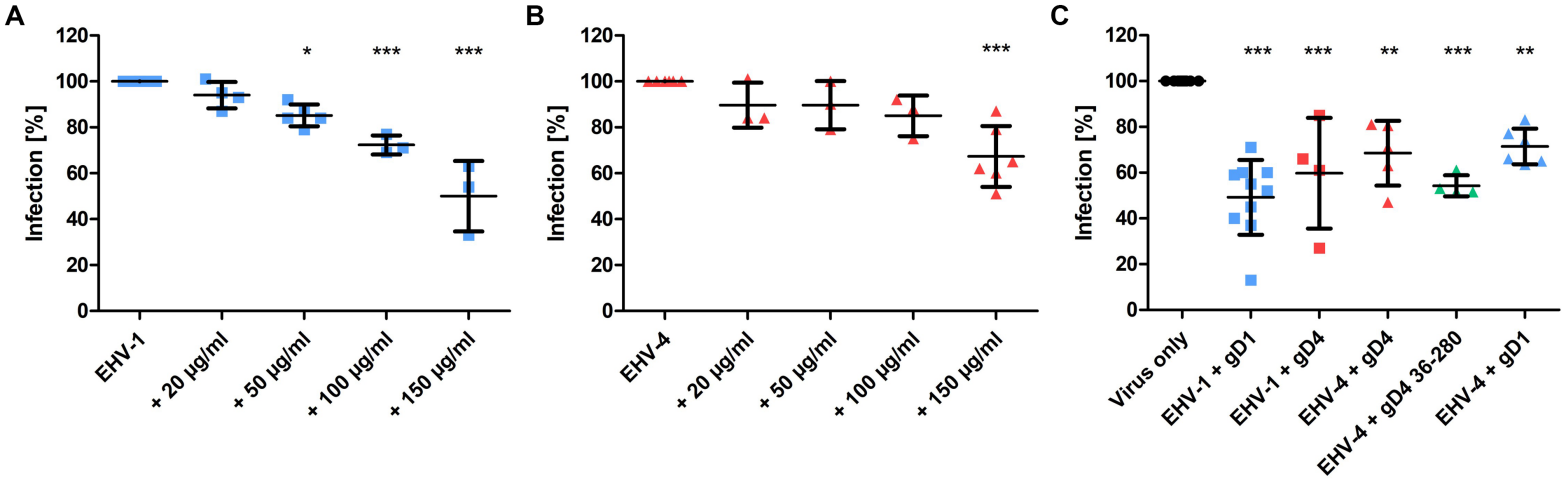
Recombinant gD1, gD4 and gD4_36-280_ blocks EHV-1 and EHV-4 infection in ED cells. **(A)** EHV-1 and **(B)** EHV-4 virus entry into ED cells blocked by different concentrations of gD1 and 4, respectively, and analyzed by flow cytometry. Cells were incubated with soluble proteins for 1 h on ice and infected with either EHV-1 or EHV-4 at MOI = 0.1. After 1 h, viruses on the cell surface were removed with citrate buffer and GFP levels were analyzed after 24–48 h by flow cytometry. **(C)** Plaque reduction assay of EHV-1 and EHV-4 with recombinant protein. ED cells were incubated for 1 h on ice with 150 μg/ml gD1, gD4 or gD4_36-280_ and infected with 100 PFU of each virus. After 1 h, viruses on the cell surface were removed with citrate buffer and cells were overlaid with methylcellulose. GFP plaques were counted after 48 h. The experiment was repeated independently three times for each protein. Plaque numbers were normalized to infection levels without recombinant proteins. Statistical analysis was done using one-way ANOVA Bonferroni’s multiple comparison test, * indicates *p* ≤ 0.05, ** indicates *p* ≤ 0.01, *** indicates *p* ≤ 0.001. Error bars represent mean with SD.

To test whether recombinant gD1 and gD4 can bind to cell surface MHC-I and subsequently inhibit virus entry, blocking assays were performed. Equine dermal (ED) cells were incubated with the recombinant proteins ranging from 0 to 150 μg/ml (0–3.5 μM) for 1 h on ice and subsequently infected with either EHV-1 or EHV-4 at a multiplicity of infection (MOI) of 0.1. Viruses expressing green fluorescent protein (GFP; Rudolph et al., 2002; Azab et al., 2009) during early infection were used to monitor and analyze the infection levels by flow cytometry. A dose-dependent reduction of infection of up to 50% and 33% on average with 150 μg/ml protein was observed for gD1 and gD4, respectively (Figures 4A,B).

Plaque reduction assays were performed by using a similar procedure. Here, 150 μg/ml of gD1, gD4, and gD4_36-280_ were used and cells were infected with 100 plaque forming units (PFU) of EHV-1 or EHV-4. In the presence of soluble gD1, plaque numbers were decreased on average by 51% for EHV-1. For EHV-4, the infection was reduced by an average of 32% after blocking the cells with soluble gD4 recombinant protein. gD4 was also able to block the entry of EHV-1 by 40%. Likewise, gD1 reduced EHV-4 infection by 29%. In general, gD1 proved to be more efficient in blocking both virus infections. The gD4 variant lacking the C-terminal membrane-proximal residues, gD4_36-280_, could inhibit EHV-4 infection more efficiently with an average of 46% and proved to be slightly more potent than untruncated gD4 (32%; Figure 4C).

Taken together, all recombinant gDs compete with viral native proteins. A dose-dependent reduction of infection was observed for gD1 and gD4. Notably, both recombinant gDs are able to efficiently block the entry of EHV-1 and EHV-4.

### 2.5. *In silico* modeling predicts gD1 and gD4 residues F213 and D261 as hot spots for MHC-I binding

No structures are available for gD1 or gD4 in complex with MHC-I. Therefore, protein–protein docking experiments were performed. Based on data from EHV-1 and EHV-4 mutational studies with diverse MHC-I genotypes it can be assumed that gD binds in close proximity to MHC-I A173 since genotypes with other residues at this position are highly resistant against infections (Sasaki et al., 2011; Azab et al., 2014). Available crystal structures of equine MHC-I Eqca-N*00602 (1.18.7-6) and Eqca-N*00601 (10.18; Yao et al., 2016) feature a glutamic acid and a threonine residue at position 173 in the α2 chain, respectively, and are known not to support EHV-1 and EHV-4 infection (Azab et al., 2014). Additionally, they contain mouse instead of equine β2m. Therefore, for *in silico* modeling of gD1 and gD4 binding MHC-I, a homology model of MHC-I genotype 3.1 was constructed to reproduce the experimental setup from the *in vitro* assays. The α-chain template (PDB ID: 4ZUU (Yao et al., 2016)) and target sequences showed 85% identity allowing the development of a high confidence model. In the next step, a homology model of equine β2m was build to achieve a physiological MHC-I state. The β2m template (PDB ID: 4ZUU) and target sequences showed 63% identity and were therefore also highly suitable for homology modeling. The final homology models of the α-chain and β2m contained no Ramachandran outliers (Ramachandran, 1963; Supplementary Figure S10). The calculated backbone RMSD to the template amounted to 0.4 Å in each structure suggesting the correct global fold of the model. All positions (101–164 and 203–259 in MHC-I and 25–80 in β2m) and geometries of disulfide bonds considered typical for MHC were correct, suggesting a high model quality. The final model structure was obtained after assembling both chains and relaxing the homology model with a molecular dynamics (MD) simulation and used directly for the gD docking (Supplementary Figure S12A).

In order to mimic the experimental setup more realistically, the peptide SDYVKVSNI was inserted into the MHC-I homology model for docking. This peptide binds in a cleft between α2 and α3 helices of MHC-I and was used in the cell-based assays. Since the peptide conformation is strongly dependent on the peptide length (Yao et al., 2016), the peptide CTSEEMNAF from MHC-I 1.18.7–6 (PDB-ID: 4ZUU) was used to build a plausible model. The modeled peptide SDYVKVSNI showed no steric clashes and exhibited reasonable bond geometries with a negligible deviation of backbone atom positions (calculated backbone RMSD of 0.8 Å to the template; Supplementary Figure S11).

To test whether additional bias emerging from peptide modeling influenced docking experiments, two docking rounds were performed. First, gD1 was docked to peptide-free MHC-I. Second, gD1 was docked to MHC-I containing the peptide SDYVKVSNI to check if docking provides comparable protein-protein interfaces (PPIs). In the first docking round, the 10 highest scored docking poses with the lowest Rosetta energy were selected (Chaudhury et al., 2011) as the initial filtering step for the peptide-free gD1—MHC-I docking. For further filtering, the rules described in the Methods section were applied (Table 3). Four out of 10 docking poses fulfilled all three rules. Subsequently, single MD simulations for each docking pose were performed to examine PPI stability. For all protein–protein complexes the backbone RMSD was calculated to obtain an overview of the amplitude of protein movements. Only one docking pose showed a nearly constant backbone RMSD value of 6 Å indicating low complex movement (Supplementary Figure S12B). In order to characterize the obtained PPI, selection criteria were applied and three residue patch classes identified in the binding surface as described in the Methods section (Table 4). An inspection of the PPI over an MD simulation trajectory with PyContact (Scheurer et al., 2018; Supplementary Table S4) revealed two gD hot spot residues: D261 (contacting binding pocket MHC-I residue R169 over the whole simulation time) and W257 (contacting binding pocket MHC-I residue I166 over the whole trajectory, Supplementary Table S4).

**Figure 5:**
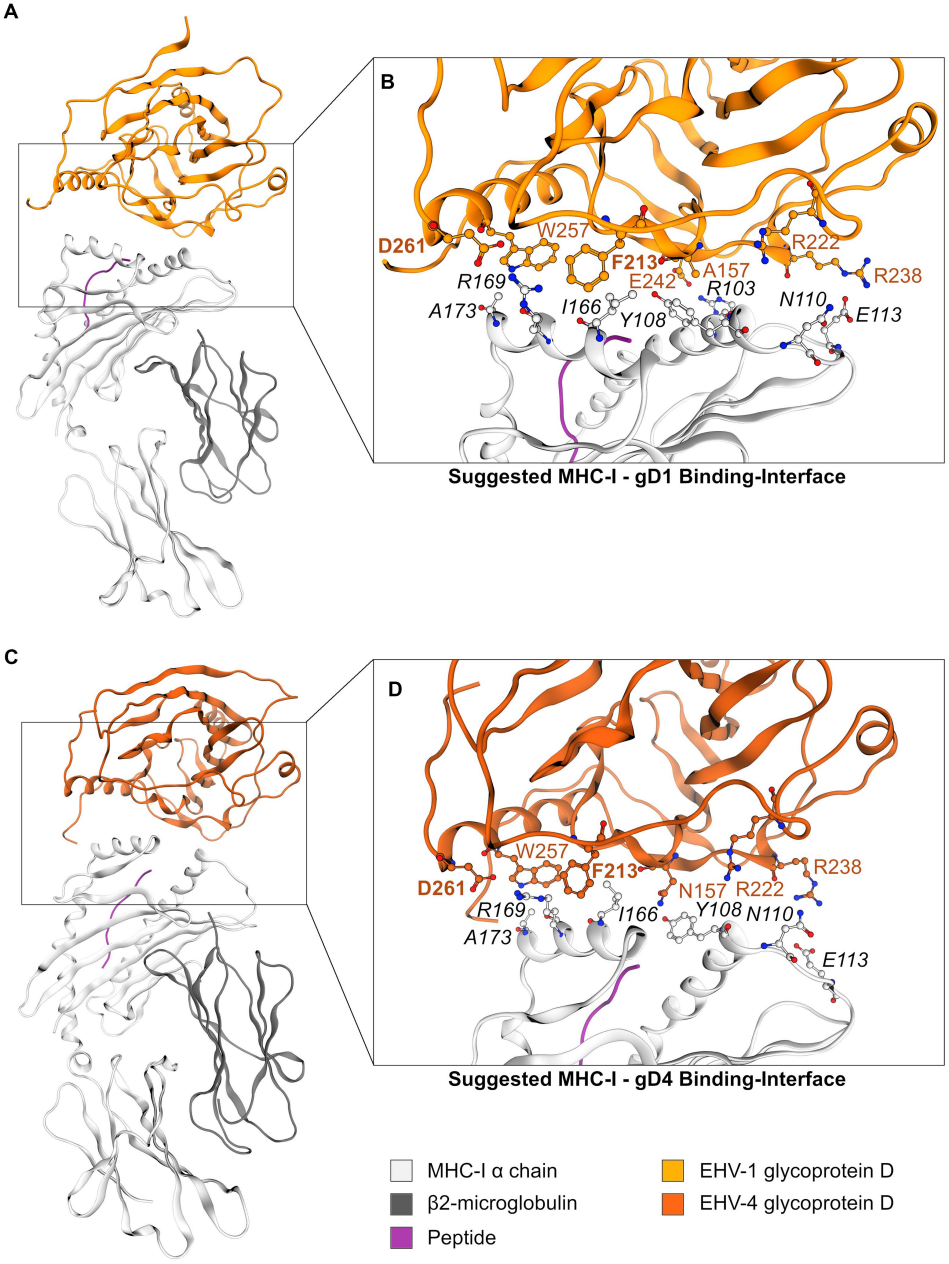
The gD1- and gD4-MHC-I interface. **(A)** Suggested model of the MHC-I—gD1 complex and **(B)** detailed view on the hypothesized binding interface. **(C)** Suggested model of the MHC-I—gD4 complex and **(D)** detailed view on the hypothesized binding interface. EHV gD residues are highlighted in orange and hot spot residues additionally in bold font. Color-code: grey ribbon—MHC-I, dark grey ribbon—β2m, orange ribbon—gD1, dark orange ribbon—gD4, purple ribbon—peptide, grey/orange balls—carbon atoms, blue balls—nitrogen atoms, red balls—oxygen atoms.

In the second round, gD1 and gD4 were docked to the MHC-I homology model with the modeled peptide under the same conditions as the peptide-free gD structures. Subsequently, a structure showing initially identified contacts between MHC-I and gD was searched. The EHV-1 gD complex was one of the 3% best scored results and the EHV-4 gD complex was in the 11% top results suggesting that both docking solutions represent low-energy protein complexes. Both structures showed similar gD-MHC-I orientations (Figures 5A,C) and recurring comparable contacts over the trajectories of MD simulations (Supplementary Table S3).

Two contacts frequently observed between gD1 and MHC-I were identified. The first hot spot residue is D261 contacting MHC-I binding pocket residue R169. The second hot spot residue is F213 contacting MHC-I binding pocket residue I166. Additionally, an extensive hydrogen bond network between residues R103—E242 and E113—R238 (Figures 5B,D) was detected (MHC-I—gD residues, respectively). All contacts and their frequencies over the trajectory of MD simulations are summarized in Supplementary Table S3. The gD1- and gD4—MHC-I PPIs over the course of MD simulations revealed minor movements measured as backbone RMSD of maximal 3.5 Å and 6.5 Å (gD1 and gD4, respectively; Supplementary Figures S12C,D).

It can be concluded that PPIs of peptide-free and peptide-bound docking poses are formed with similar residue patches (Supplementary Tables S3, S4) suggesting that the presence of the peptide in MHC-I does not influence gD binding. We observed that peptide-bound docking poses exploit larger PPIs with more possible interactions than the peptide-free docking pose. We assume that more contacts between gD and MHC-I are favorable for the binding. Therefore, peptide-bound docking poses were chosen as the final ones. Based on the optimized docking models, two gD variants were designed for experimental validation in the next step: F213A and D261N. Both residue exchanges are predicted to disrupt gD—MHC-I contacts and lead to inhibition of viral replication in a cell-based assay.

### 2.6. Mutating F213A and D261N in EHV-1 and 4 gD leads to growth defects

The gD1/4-MHC-I binding hypotheses (Figure  5) were experimentally investigated by mutating the proposed key residues F213 to alanine and D261 to asparagine in EHV-1 and EHV-4 gDs. Two-step Red-mediated mutagenesis (Tischer et al., 2006) was performed on EHV-1 and EHV-4 bacterial artificial chromosomes (BACs) and multi-step growth kinetics with plaque reduction assays were used for virus characterization.

**Figure 6:**
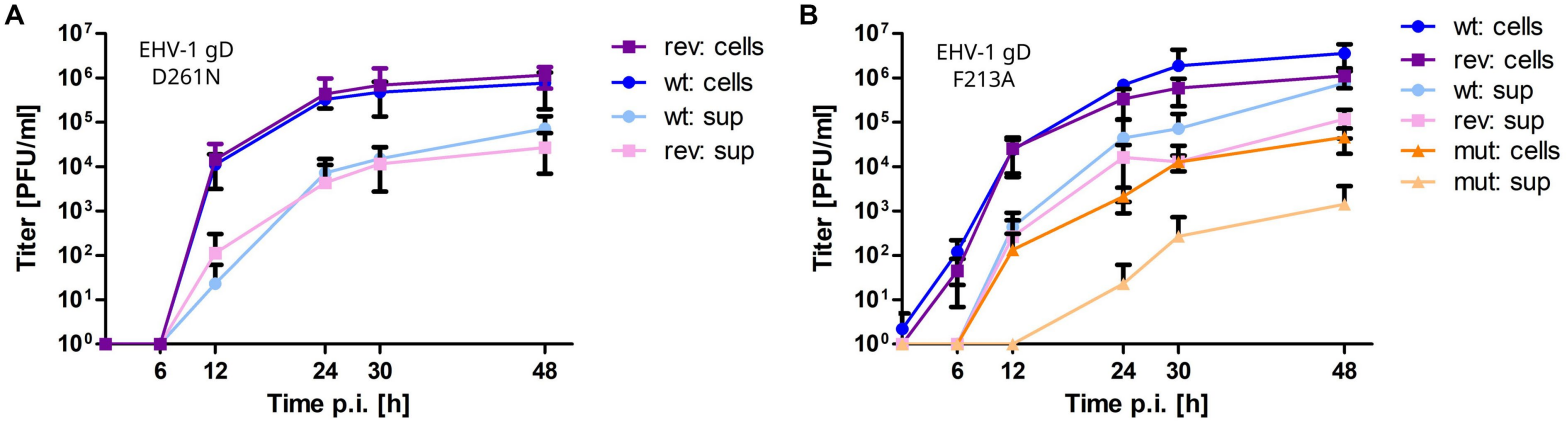
Mutating gD1 residues D261N and F213A impairs EHV-1 growth in ED cells. Multi-step growth kinetics of EHV-1 parental virus and gD mutants. ED cells were infected with an MOI of 0.01, cells and supernatant were collected separately at indicated time points post infection and titrated on ED cells. Shown are means with standard deviation (SD) of three independent experiments. **(A)** EHV-1 parental virus (blue colors) and EHV-1-gD_D261N_ (violet colors). **(B)** EHV-1 parental virus (blue colors), EHV-1-gD_F213A_ (orange colors) and EHV-1-gD_F213A_ (violet colors).

All mutant viruses were successfully reconstituted from mutated BAC and the modified gD gene sequences were confirmed by Sanger sequencing. EHV-1-gD_F213A_ displayed a significant 2-log reduction in growth kinetics and low titers in cell supernatant compared to the parental virus. Reverting the mutation rescued virus growth in cell culture (Figure 6B). Plaque sizes of wild type, mutant and revertant viruses were similar. The virus mutants EHV-1-gD_D261N_, EHV-4-gD_D261N_ and EHV-4-gD_F213A_ did not grow in cells to the extent where growth kinetics could be analyzed. However, reverting the residue exchange in EHV-1-gD_D261N_ rescued virus growth (Figure 6A). Taken together, the gD_D261N_ and gD_F213A_ variants lead to replication-deficient viruses in EHV-1 and EHV-4.

## 3. Discussion

Although details of cell entry of alphaherpesviruses can differ greatly between virus species, four common steps characterize the whole entry processes. First gB and/or gC attach in a relatively unspecific and reversible manner to cell surface heparan sulfate proteoglycans (HSPG) and chondroitin sulfate proteoglycans (CSPG; Osterrieder, 1999; Spear and Longnecker, 2003; Azab et al., 2010). This charge-based interaction is stabilized by a stronger and specific receptor-ligand interaction (Csellner et al., 2000) followed by a signaling cascade which is activated by gD and gH/gL (Azab et al., 2012). The latter process leads ultimately to the fusion of the viral envelope with the cell membrane or in some cases to entry via endocytosis through gB (Azab et al., 2015; Azab and Osterrieder, 2017), gD is the essential protein that, in case of EHV-1 and EHV-4, binds to equine MHC-I (Kurtz et al., 2010; Sasaki et al., 2011; Azab et al., 2014). The mode of gD binding to MHC-I remains elusive, although the structural understanding of alphaherpesviral gDs binding to their putative receptors has been largely extended in the last years (Carfi et al., 2001; Krummenacher et al., 2005; Li et al., 2017; Yue et al., 2020). Here, we present crystal structures of EHV-1 and EHV-4 gDs and propose a binding model to equine MHC-I through the key residues F213 and D261.

The crystal structures of EHV-1 and EHV-4 gDs revealed an IgV-like fold with large N- and C-termini wrapping around the core which is common for members of the gD polypeptide family. Despite high variability in sequence identities, the overall structure of alphaherpesviral gDs is conserved with only small variations in the loop regions and number of helices (Carfi et al., 2001; Krummenacher et al., 2005; Li et al., 2017; Yue et al., 2020; Figure 2; Supplementary Figure S8). The gD termini have been shown to be important for the entry process in HSV-1 (Krummenacher et al., 2005). To allow receptor binding, the C-terminus needs to be displaced to free the N-terminal binding site. This could be a mechanism to prevent early onset of the fusion process before the ligand and receptor are in proximity. Subsequently, the displacement of the C-terminus allows the formation of an N-terminal hairpin loop that is crucial for HVEM binding, since exclusively gD N-terminal residues (7–15 and 24–32) interact with the receptor (Carfi et al., 2001; Krummenacher et al., 2005; Lazear et al., 2008). The displacement of the C-terminal tail is also needed for the complex formation with nectin-1 as the binding sites overlap with those of HVEM with additional C-terminal interactions (residues 35–38, 199–201, 214–217, 219–221, 223; Di Giovine et al., 2011). The formation of an N-terminal loop is not involved in nectin-1 binding since the deletion of residues 7–32 had little impact on the interaction (Manoj et al., 2004). The N-terminus of gD1 and gD4 is, similarly to PrV gD, shorter than in HSV-1 gD, suggesting that HVEM cannot function as an entry receptor in these viruses. In fact, it has already been experimentally observed that HVEM is not used as entry receptors by PrV (Li et al., 2017). Similarly, we observed that EHV-1 also does not employ the equine HVEM homolog either (Azab and Osterrieder, 2017). In HSV-1, gD forms a dimer in the unbound state on the virus envelope (Handler et al., 1996). This is thought to stabilize the C-terminus since viruses with a destabilized terminus could not efficiently enter cells. Although the ionic contact and high Complex Formation Significance Score of the here solved EHV-1 gD dimer interface suggest a similar function, no dimer was observed in SEC, SEC-MALS, and MS analysis. Therefore, we suppose that gD1 has no dimeric form on the virus envelope.

In contrast to results from C-terminally truncated gD homologs, which display a dramatic increase in receptor affinity (Table 2), truncated gD4_36-280_ binds MHC-I similarly as the non-truncated version. The higher affinities in the truncated homologs are explained in the literature by a faster interaction with the receptors, since the C-terminus that blocks the binding site is not required to be displaced upon binding anymore (Krummenacher et al., 2013). That the C-terminal truncation had no significant effect on the receptor affinity of EHV-4 gD suggests that the mode of binding differs from other alphaherpesviruses, however, the role of the C-terminus remains to be determined. Taking into account that EHV-1 and EHV-4 bind to MHC-I instead of HVEM or nectin-1, a different binding mechanism would be assumed. In line with results obtained from gD4_36-280_, truncated BoHV-1 gD interaction with nectin-1 showed no increased receptor affinity (Yue et al., 2020; Table 2). A conformational change in the loop region between the G-strand and α2 helix is needed for receptor binding (Yue et al., 2020) and might explain why the affinity of the truncated BoHV-1 gD does not increase.

SPR analysis showed binding of recombinant gD1, gD4, and gD4_36-280_ to recombinant MHC-I with micromolar affinities. The $K_{d}^{\mathrm{app}}$ are higher than in gD homologs of HSV-1, HSV-2, and PrV binding nectin-1. However, HSV-1 gD binding to HVEM displays similar affinities (Table 2 upper part). Nevertheless, there are two limitations of the SPR analysis in this study. First, the MHC-I molecule Eqca-1*00101 (3.1) used here allowed lower infection rates in a previous study than the molecule Eqca-16*00101 (2.16; Azab et al., 2014). Due to construct design reasons, the gene 3.1 fitted the purpose of crystallography better, although, no crystal structure could be obtained. However, the 2.16 molecule should display higher receptor affinities than the one observed in this study. Second, the linker region (GGGSGGGSGGGS), inserted to tether the peptide in the MHC-I complex to the β2m C-terminus, might interfere with gD receptor binding. Our attempt to model the linker to the MHC-I molecule that binds gD1 in the position hypothesized here supports this hypothesis. Nevertheless, the results from blocking assays confirm that the receptor affinities of soluble gDs are unlikely to be in the nanomolar range since relatively high concentrations were necessary to see an effect on virus entry. Furthermore, blocking assays revealed that gD1, gD4, and gD4_36-280_ can block cell surface MHC-I and thus compete with native gD in the viral envelope. It could be demonstrated that gD1 can block EHV-4 infections and vice versa implying that the receptor interaction is very similar in both viruses. This finding is supported by the binding models presented here.

The finding in SPR analysis that the C-terminally truncated gD4 does not display an increased receptor affinity was confirmed in blocking assays, thus suggesting that the receptor-binding mode differs from HSV and PrV, which is not surprising as they enter through different receptors.

The proposed docking position of gD1 to MHC-I explains why MHC-I Eqca-16*00101 (2.16) allows higher infection rates than Eqca-1*00101 (3.1; Azab et al., 2014). The residue 103 in the 3.1 α1 region is an arginine, which is more spacious than asparagine in 2.16, thus preventing a closer interaction with gD and leading to lower receptor binding affinities. A binding hypothesis with MHC-I 2.16 and a crystal structure of this molecule could confirm that theory. A173 of MHC-I has been shown previously to play a major role in the entry of EHV-1 and EHV-4 by two studies. First, the entry of EHV-1 into usually non-susceptible NIH3T3 cells transfected with altered hamster MHC-I Q173A has been shown together with the negative effect on infection rates of hydrophilic residues at position 173 in equine MHC-I (Sasaki et al., 2011). Second, it has been demonstrated that not all equine MHC-I genes support entry of EHV-1 and 4 into equine MHC-I transfected mouse mastocytoma (P815) cells and that MHC-I genes harboring residues other than alanine at position 173 are highly resistant against EHV-1 and 4 infections (Azab et al., 2014). The gD1/4-MHC-I binding hypotheses explain the role of MHC-I A173 well by showing that bulkier amino acids at that position lead to steric hindrance in the gD binding pocket. This applies to MHC-I alleles 3.3 (V173), 3.4 (T173), 3.5 (E173), and 3.6 (V173), which do not support EHV-1 and 4 entry (Sasaki et al., 2011; Azab et al., 2014). The model can even explain why the genotype Eqca-7*00201 (3.7), although harboring an alanine at position 173, does not allow entry of EHV-1 and 4 into P815 3.7 (Azab et al., 2014). The glutamine residue at position 174 is assumed to hinder gD binding sterically. The side-chain would point in the bound state into a hydrophobic residue-patch (W253, F256, W257) of gD, leading to an enthalpic penalty. However, the inability of the viruses to enter via the MHC-I haplotype Eqca-2*00101 (3.2) which harbors A173 and A174 cannot be explained by the binding model. The topology of this MHC-I molecule is predicted to be very similar to those allowing virus entry. A crystal structure of the 3.2 MHC-I gene might give an explanation. Mutations in the gD binding pocket R43, W253, F256, and W257 could prove useful for a more detailed evaluation of the predicted interaction with MHC-I A173. Furthermore, the here presented binding position of MHC-I to gD leaves open the proposed gH/gL interface (Cairns et al., 2019).

Another observation by Sasaki et al. (2011) was that the mutation W171L in equine MHC-I impairs virus entry into NIH3T3 cells transfected with this MHC-I. Although the cell surface expression of this mutant was reduced, this is still interesting since structural data show that W171 points towards the peptide in the binding groove and should therefore not be involved directly in binding gD. The tryptophan would be able to stabilize some peptides with hydrogen bonds, whereas a leucine would not. A leucine at position 171 could therefore lead to a more loosely bound peptide with a higher flexibility, resulting in an interference via the gD-MHC-I binding. This theory would suggest that the peptide in the MHC-I binding groove itself could play a role in the receptor-ligand interaction, which could be tested by using different peptides bound to MHC-I in blocking assays and by testing mutated equine MHC-I W171L in blocking assays with soluble recombinant gDs.

Considering all these results, the question arises whether EHV-1 and EHV-4 can facilitate entry through, so far, unknown non-equine MHC-I molecules. Sasaki et al. (2011) demonstrated that mutated hamster MHC-I Q173A allowed low EHV-1 infection. Unfortunately, EHV-4 has not been tested in the same manner. A computational approach could be employed to search for non-equine MHC-I molecules that are similar in the binding region that is visible in the gD1/4-MHC-I binding model and be used to select promising targets for transfection/infection assays.

Experimentally, EHV-1 and EHV-4 infections could be tested in cell lines from susceptible species, e.g., bovine, rabbit, monkey, pig, cat, human (Studdert and Blackney, 1979; Ahn et al., 2010), alpacas, lamas, polar bears (Greenwood et al., 2012), and rhinoceros (Greenwood et al., 2012; Abdelgawad et al., 2014, 2015) cell lines, with and without inhibited MHC-I expression by using β2m knockdown as in Sasaki et al. (2011).

Taken together, the proposed docking modes of gD1 and gD4 to MHC-I can explain several experimentally obtained results and are therefore plausible. Additionally, the docking models are supported by EHV-1 and EHV-4 viruses with mutated gD_F213A_ and gD_D261N_ that all exhibited an impaired growth. There are two limitations in this experiment. Firstly, it was very difficult to revert EHV-4 mutations to original status to confirm that the observed effect was exclusively due to the gD_F213A_ and gD_D261N_ variants. Secondly, additional experiments are needed to exclude that the observed effect of the mutations is due to reduced binding to gH/gL. Recombinant protein harboring the same mutations as the viruses could be used in SPR to test if gD binding is impaired. Nevertheless, the present results show that the gD residues F213 and D261 play a key role during entry of EHV-1 and EHV-4 providing starting points for further mutational studies possibly leading to an efficient vaccine and might also be used to generate gD-based EHV-1 and EHV-4 inhibitors for reduction of clinical symptoms in horses and non-definite hosts.

## 4. Materials and methods

### 4.1. Viruses

EHV-1 strain RacL11 and EHV-4 strain TH20p are maintained as bacterial artificial chromosome infections clones (BAC). The viruses have GFP under the control of the HCMV major immediate-early (IE) promoter inserted into the Mini-F sequence to easily recognize infected cells. Clones were generated as described previously in Rudolph et al. (2002) and Azab et al. (2009, 2011). The viruses were grown on equine dermal (ED) cells (CCLV-RIE 1222, Federal Research Institute for Animal Health, Greifswald, Germany) at 37°C under a 5% CO_2_ atmosphere.

### 4.2. Cells

ED cells were grown in Iscove’s modified Dulbecco’s medium (IMDM; Pan, Biotech, Aidenbach, Germany) containing 20% fetal bovine serum (FBS; Biochom GmBH, Berlin, Germany), 1 mM sodium pyruvate (Pan Biotech, Aidenbach, Germany), 1% nonessential amino acids (NEAA; Biochom GmBH, Berlin, Germany), and P-S solution (100 U/ml penicillin: Panreac, AppliChem GmBH, Darmstadt, Germany); 100 μg/ml streptomycin: Alfa Aesar, Thermo Fisher Scientific, Kandel, Germany (P-S) at 37°C under a 5% CO_2_ atmosphere.

Human embryonic kidney (293 T, ATCC CRL-11268) cells were propagated in Dulbecco’s modified Eagle’s medium (DMEM; Biochom GmBH, Berlin, Germany), supplemented with 10% FBS and P-S. Sf9 cells (IPLB-Sf21-AE, Invitrogen, Germany) were propagated in serum free Sf-900 III medium (Gibco, Thermo Fisher Scientific, New York, USA) and High Five™ cells (BTI-TN-5B1-4, Invitrogen, Germany) in serum free Express Five medium (Gibco, Thermo Fisher Scientific, New York, USA) at 27°C on orbital shaker.

### 4.3. Construction of expression plasmids

**Figure 7:**
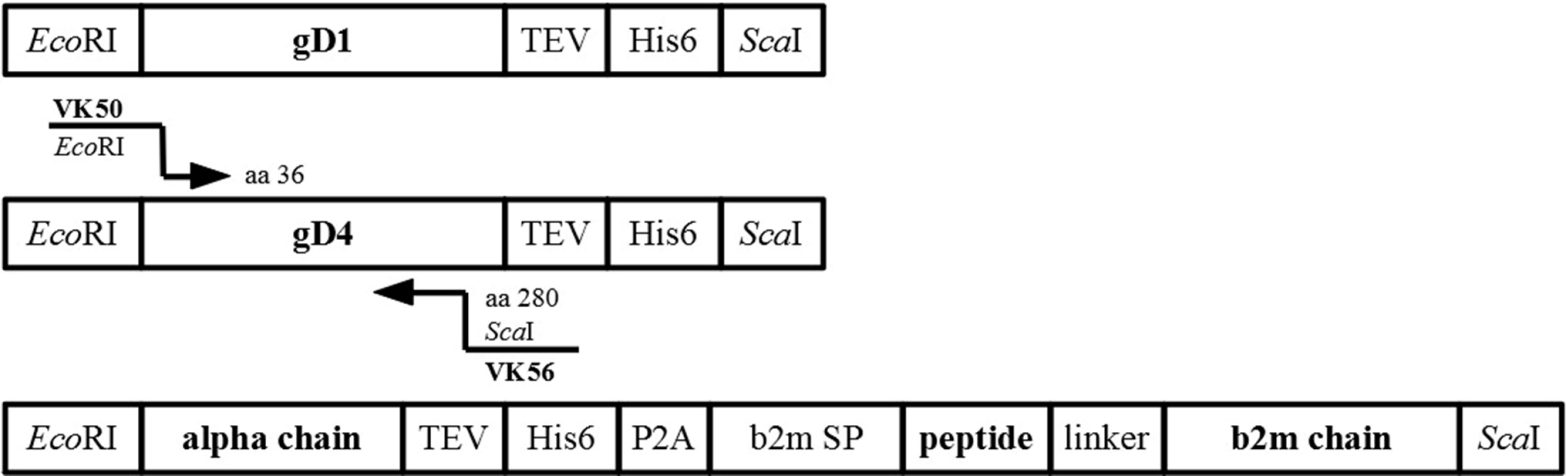
Synthetic genes used for cloning. Schematic representation of synthetic genes for protein production of gD1, gD4, equine MHC-I 3.1 with cloning strategy for gD4_36-280_.

Constructs were amplified from insect cell codon-optimized DNA fragments (Bio Basic Inc., Canada) for protein production in High Five insect cells. Synthetic truncated genes contained the gene of interest (gD1 residues 32–249, gD4 residues 32–249, equine MHC-I 3.1,), a C-terminal TEV protease cleavage site (ENLYFQG), and a hexa-histidine tag (His_6_), all flanked by *EcoRI-and Sca*I-restriction sites (Figure 7). Sequences of codon optimized genes can be found in the supplementary data. A further truncated form of gD4 containing the residues 36–280 was amplified from gD4 synthetic gene with the primer pair VK50/VK56 (Supplementary Table S2).

The *Autographa californica* nuclear polyhedrosis virus (AcNPV) baculovirus gp64 signal sequence under control of the very late polyhedrin promoter was inserted into the insect cell vector plasmid pACEBac1 (Addgene, LGC Standards Teddington, UK) by using another synthetic gene (VK18, LGC Genomics, Berlin, Germany) and the primer pairs VK7/VK7 (Supplementary Table S2). Subsequently, plasmids containing synthetic genes (gD1, gD4, MHC-I) were amplified in *Escherichia coli* (*E*. *coli*), purified, and digested with *EcoRI-and Sca*I-restriction enzymes for insertion into the transfer vectors which was digested with the same restriction enzymes. After ligation, these plasmids were transformed into DH10MultiBac electrocompetent cells and recombinant baculoviruses produced according to manufacturer’s instructions (Bac-to-Bac expression system, Invitrogen). All constructs were verified by sequencing (VK8 or VK10/WA2, VK35/38; Supplementary Table S2). Recombinant BACs were isolated and used for virus production in Sf9 cells as described in Santos et al. (2012).

### 4.4. Protein production and purification

Equine MHC-I, gD1_32-349_, gD4_32-349_, and gD4_36-280_ were expressed in HighFive cells. Cell supernatant was harvested after 72 h post infection by centrifugation. The pH was adjusted to 7 with 1 M tris (hydroxymethyl) aminomethan (Tris)-HCl buffer at pH 9 on ice and incubated for at least 1 h with washed Ni^2+^-NTA beads for affinity chromatography. Beads with bound recombinant protein were collected by a gravity flow column and the proteins were eluted with a buffer containing 20 mM Tris–HCl at pH 7.5 or 2-(N-morpholino) ethanesulfonic acid (MES) at pH 6 for gDs and MHC-I, respectively, and 200 mM NaCl, 5% (v/v) glycerol, and 200 mM imidazole. Concentrated protein was loaded onto a 16/600 Superdex 200 gel filtration column (GE Healthcare Piscataway, NJ). The buffer conditions were the same as in Ni^2+^-NTA affinity chromatography but with 20 mM NaCl and no imidazole. Proteins collected from size-exclusion chromatography were concentrated (Concentrators, Amicon Ultra, Millipore, Darmstadt, Germany), aliquoted and directly used for crystallization or stored at *−*80°C.

### 4.5. Crystallization, structure determination, and refinement

Crystals of EHV-1 gD were obtained by the sitting-drop vapor-diffusion method at 18°C with a reservoir solution composed of 0.1 M Tris/HCl buffer at pH 8.5, 0.2 M MgCl_2_, and 30% (w/v) polyethylene glycol (PEG) 4,000. Crystals were cryo-protected with a solution composed of 75% mother liquor and 25% (v/v) glycerol and subsequently flash-cooled in liquid nitrogen. Synchrotron diffraction data were collected at the beamline P11 at DESY (Hamburg, Germany) and at the beamline 14–2 of the MX beamline of the BESSY II (Berlin, Germany) and processed with X-ray detector software (XDS; Kabsch, 2010). The structure was solved by molecular replacement with PHASER (Bunkóczi et al., 2013) using the coordinates of PDB-ID 2C36 as search model for gD1 which was then used as search model for gD4. A unique solution with two molecules in the asymmetric unit for gD1 and molecule for gD4 were subjected to the program AUTOBUILD in PHENIX (Adams et al., 2010) and manually adjusted in COOT (Emsley et al., 2010). The structures were refined by maximum-likelihood restrained refinement using PHENIX (Adams et al., 2010; Afonine et al., 2012). Model quality was evaluated with MolProbity (Williams et al., 2018) and the JCSG validation server (Yang et al., 2004). Secondary structure elements were assigned with DSSP (Kabsch and Sander, 1983) and for displaying sequence alignments generated by ClustalOmega (Sievers et al., 2011) ALSCRIPT (Barton, 1993) was used. Structure figures were prepared using PyMOL (DeLano, 2002). Coordinates and structure factors have been deposited in the PDB for gD1 with PDB-ID 6SQJ as well as for gD4 with PDB-ID 6TM8. Diffraction images have been deposited at proteindiffraction.org (gD1: DOI 10.18430/m36sqj and gD4 DOI 10.18430/m36tm8).

### 4.6. Mass spectrometry analysis

Intact protein mass of gD1, gD4, and MHC-I was determined by matrix-assisted laser desorption ionization-time of flight mass spectrometry (MALDI-TOF-MS) using an Ultraflex-II TOF/TOF instrument (Bruker Daltonics, Bremen, Germany) equipped with a 200 Hz solidstate Smart beam™ laser. Samples were spotted using the dried-droplet technique on sinapinic acid (SA) or 2,5-dihydroxybenzoic acid (DHB) matrix (saturated solution in 33% acetonitrile/0,1% trifluoroacetic acid). The mass spectrometer was operated in the positive linear mode, and spectra were acquired over an m/z range of 3,000-60,000. Data was analyzed using FlexAnalysis 2.4. software provided with the instrument.

Protein identity was determined by tandem mass spectrometry (MS/MS) of in-gel digested Coomassie stained protein with 12_,_5 μg*_/_* ml Glu-C and trypsin, and 10 μg*_/_* ml Asp-N in 25 nm ammonium bicarbonate.

N-terminal c and C-terminal (z + 2) sequence ion series were generated by in-source decay (ISD) with 1,5-diaminonaphthalene (1,5-DAN) as matrix (20 mg/ml 1,5-DAN in 50% acetonitrile/0,1% trifluoroacetic acid). Spectra were recorded in the positive reflector mode (RP PepMix) in the mass range 800–4,000.

### 4.7. SEC-MALS analysis

For molecular mass determination of soluble, recombinant gD1, SEC-MALS (Tarazona and Saiz, 2003) was performed. Protein solution was run at room temperature on a Superdex 75 10/300 Gl (GE Healthcare, Piscataway, NJ) column with 2 mg*/*ml gD1 and a mobile phase composed of Tris–HCl at pH 7.5, 200 mM NaCl, 5% glycerol, and 0.02% sodium azide, attached to a high-performance liquid chromatography (HPLC) system (Agilent Technologies, Santa Barbara, CA, USA) with a mini DAWN TREOS detector (Wyatt Technology Corp., Santa Barbara, CA, USA). Data was acquired and analyzed with ASTRA for Windows software package (version 6.1.2).

### 4.8. Surface plasmon resonance

Binding kinetics of soluble gD1, gD4, and gD4_36-280_ binding to amine-coupled recombinant, equine MHC-I 3.1 were measured at 25°C on a surface plasmon resonance (SPR) GE Biacore J Biomolecular Interaction Analyser instrument (Uppsala, Sweden) using a polycarboxylate hydrogel sensor chip HC200M (XanTec bioanalytics GmbH). The second channel was coated with poly-L-lysine and positive nanogels (size 214 nm; Dey et al., 2018) that were shown to interact only weakly with gDs and used as negative control. The control sensorgrams were subtracted from reaction sensorgrams and normalized. The surfaces were regenerated with buffer containing 200 mM NaCl and 10 mM NaOH after each cycle. Serial dilutions of gDs ranging from 0 to approximately 10 μM were injected at medium flow and the interaction with MHC-I was monitored for 15 min to quantify the equilibrium response $R_{\mathrm{eq}}$ . In the cases in which a clear flattening of the sensorgramm could not be observed within the experimental time, $R_{\mathrm{eq}}$ was extrapolated using a simple exponential function (see Supplementary Figure S13 for an example). The response curves of gDs binding to the MHC-I were fitted to the binding model $R_{\mathrm{eq}}=\frac{R_{\infty }\left[A\right]}{K_{d}^{\mathrm{app}}+\left[A\right]}$ (Schasfoort, 2017) using Sigma plot 12.0 software. $R_{\infty }$ is the maximum signal corresponding to saturation, $\left[A\right]$ is the protein concentration and $K_{d}^{\mathrm{app}}$ is the apparent dissociation constant.

### 4.9. Generation and analysis of gD1/4-MHC-I binding model

#### 4.9.1. Protein data

Sequences of MHC-I and β2m were obtained from UniProt-Databank (UniProt Consortium). The protein sequences with their respective UniProt IDs are listed in Table 3.

**Table 3:** Protein structures obtained from UniProt with their respective IDs.

Protein	UniProt ID
MHC-I gene Eqca-1*00101	Q30483
MHC-I genotype Eqca-N*00602	Q860N6
Horse β2m	P30441
Mouse β2m	P01887

#### 4.9.2. Homology modeling

Homology models were prepared using MOE (version 2018.0101; Molecular Operating Environment, Chemical Computing Group ULC, Montreal, Canada). The models were constructed using GB/VI scoring (Labute, 2008) with a maximum of 10 main chain models. To check geometry of obtained homology models, Ramachandran (phi-psi angle) plots (Ramachandran, 1963) were calculated with MOE.

The full MHC-I gene 3.1 model was prepared based on the hybrid equine α-chain—mouse β2m X-ray crystal structure with the best resolution [PDB-ID: 4ZUU (Yao et al., 2016)]. The α-chain and β2m homology models were superposed onto the template. All side chain clashes were removed by energy minimization using the OPLS-AA force field (Kaminski et al., 2001), resulting in the full MHC-I gene 3.1 model. The complete model was relaxed in an MD simulation on settings described below.

#### 4.9.3. Protein–protein docking

MHC-I-gD1 complex was prepared with MOE 2018 by protonation (Labute, 2009), modeling of missing side chains, deleting water molecules and charging termini. Protein–protein docking was performed using Rosetta 3 suite (version 2018–33; Gray et al., 2003; Sircar et al., 2010). The orientations of MHC-I and gD were randomized (flags-randomize 1-randomize 2) and spun (flag-spin) to the beginning of the docking process. Docking perturbation parameters were set to default: 3 Å translational and 8° rotational movement (flag-dock_pert 3 8; Chaudhury and Gray, 2008). The residue side chains of both docking partners were allowed to rotate around the *χ*^1^ and the *χ*^2^ angles (flags-ex1-ex2). In total 10,000 docking runs were conducted (flag-nstruct 10,000) as recommended by the Rosetta documentation (JBBM et al., 2017), yielding over 7,000 poses in each docking round. A flat harmonic distance constraint between the Cα of MHC-I A173 and the gD backbone was added based on reported genotype studies indicating the pivotal role of MHC-I A173 (Sasaki et al., 2011; Azab et al., 2014). This allowed us to limit the number of possible protein–protein docking complexes and perform local docking as recommended by the Rosetta documentation (JBBM et al., 2017). Constraint parameters were set to the default (JBBM et al., 2017): Distance 0 Å, standard deviation 1 Å and tolerance 5 Å to achieve the closest possible proximity between chains. In order to obtain a full MHC peptide complex, peptide SDYVKVSNI, as used in cell-based assays, was manually fitted into the MHC-I cleft. To fit our sequence, the peptide structure from the template (PDB-ID: 4ZUU) containing a nonapeptide CTSEEMNAF was superposed on the MHC-I homology model. The peptide sequence was manually mutated. The side chain conformations were adjusted using MOE’s rotamer tool and energy minimized using the OPLS-AA force field to relax atomic clashes.

Finally, gD1 and gD4 were docked into the prepared MHC–peptide complex. In order to find the final and most plausible docking pose of gD1 and gD4 in complex with MHC-I-peptide, an in-house developed MD Analysis-based script (version 0.19.2; Michaud-Agrawal et al., 2011; Gowers et al., 2019) was used to find ionic key-contact defined as a distance of maximal 4.5 Å between C*γ* atom of D261 in gD and C*ζ* atom of R169 in MHC-I. The script was run in a Python 3.6 environment (van Rossum, 1995).

#### 4.9.4. Filtering of docking poses and classification of residues involved in the protein–protein interface

In order to filter the most plausible from all best-scored docking poses, we applied three rules based on reported statistical evaluation of residue probability-distribution in various protein–protein interfaces based on alanine scan studies and crystallization experiments (Bogan and Thorn, 1998; Liu and Li, 2010) and biological function of Herpesvirus gD (Table 4; Zhang et al., 2011). The residues involved in the protein–protein binding in the obtained binding hypotheses were classified according to the O-ring theory (Table 5; Bogan and Thorn, 1998).

**Table 4:** Criteria applied for analysis of protein–protein interfaces to filter the most energetically favored docking poses.

Filtering criterion	Rationale
Discarding docking poses with the C-terminus participating in the resulting PPI	Orientation unlikely to be correct because the C-terminus merges with a transmembrane-helix anchoring in viral membrane (Zhang et al., 2011)
Discarding docking poses without lipophilic residues in the modeled PPI	Protein–protein interfaces with lipophilic contacts are common and entropically favored according to Liu and Li, (2010)
Accepting poses with contact to residues R, D, H, I, K, P, W, Y buried in interface areas	These residues are statistically enriched in protein–protein binding interfaces according to the O-ring theory (Bogan and Thorn, 1998)

**Table 5:** Classification of residues applied for analysis of protein–protein interface.

Residue type in the binding interface	Definition
Hot spot residues	Amino acids statistically enriched in binding sites of protein–protein complexes and contributing more than 2 kcal/mol to the binding energy (Bogan and Thorn, 1998) or form lipophilic contacts (Liu and Li, 2010)
O-ring	Residues preventing solvation of binding hot spots (Bogan and Thorn, 1998)
Binding pocket	Counterpart of hot spot residues on the surface of the binding partner

#### 4.9.5. Molecular dynamics simulations and protein–protein interaction analysis

Molecular Dynamics (MD) simulations were prepared using Maestro (version 11.7; Schrödinger, New York, USA) and carried out using Desmond 2018–3 (version 5.5; Bowers et al., 2006). All systems were simulated on water-cooled GeForce RTX 2080 Ti graphics processing units (NVIDIA Corporation, Santa Clara, USA). The full MHC-I gene 3.1 homology model was solvated in a cubic box with 12 Å buffering with the SPC water model (Toukan and Rahman, 1985). The system was neutralized using sodium or chloride ions and osmotic pressure was adjusted with 0.15 M sodium chloride to achieve an isotonic system. The subsequent system relaxation was performed according to the default Desmond protocol. The MD simulation ran under periodic boundary conditions and as an NPT ensemble (constant particle number, pressure and temperature) using the OPLS 2005 force field (Banks et al., 2005). The MD simulation was performed in one replicate over 100 ns. Coordinates of the relaxed model were retrieved after the backbone RMSD (Supplementary Figure S12A) had reached a stable plateau around 3 Å indicating protein equilibration.

Docking poses were simulated under the same conditions as the homology models. The movement of protein–protein complex hypotheses was observed in a single MD simulation over 100 ns resulting in approx. 5,000 complex conformations. MD simulations of the final selected docking pose were performed in triplicates using different seeds. The simulated systems contained around 140,000–168,500 atoms. The proteins were wrapped, aligned on the backbone and visually inspected in VMD (Humphrey et al., 1996; version 1.9.3). Protein–protein interactions were analyzed using PyContact (Scheurer et al., 2018; version 1.0.1) on default settings (distance cutoff 5.0 Å, angle cutoff 120.0° and distance cutoff between hydrogen and hydrogen bond acceptor of 2.5 Å). The PyContact analysis was run in a Python 2.7 environment (van Rossum, 1995).

### 4.10. BAC mutagenesis

The point mutations F213A and D261N in EHV-1 and EHV-4 gDs were introduced via a two-step Red recombination (Tischer et al., 2006). In brief, polymerase chain reaction (PCR) primers (Supplementary Table S2) were designed in a way that the 50 nucleotide recombinantion arms include the point mutation and sequence to amplify the *kan*^R^ gene. For construction of EHV-1-gD_D261N_, EHV-1gD_F213A_, EHV-4-gD_D261N_, and EHV-4-gD_F213A_ the primer pairs VK61/VK62, VK63/VK64, VK65/VK66, and VK67/VK68 were used for PCR amplification, respectively. After Dpn-1 digest of PCR products, fragments were electroporated into GS1783 containing EHV-1 or EHV-4 BACs. DNA from Kanamycin resistant colonies was extracted and correct mutants were selected based on Restriction fragment length polymorphism (RFLP) using the restriction enzyme Pst-I. Correct clones were subjected to another round of Red recombination to remove the *kan*^R^ gene. Final clones were further analyzed by RFLP and sequencing, BAC extracted, purified and transfected into 293 T cells. Cells and supernatant were harvested 3 days post transfection and used to infect ED cells. Revertants were produced from mutant clones using the same procedure with primer pairs VK69/VK70, VK71/VK72, VK73/VK74, and VK75/VK76 for producing EHV-1R-gD_D261N_, EHV-1R-gD_F213A_, EHV-4R-gD_D261N_, and EHV-4-RgD_F213A_, respectively. All genotypes were confirmed by PCR, RFLP, and Sanger sequencing using the primer pair WA2/VK8 and WA2/VK10 (Supplementary Table S2) for EHV-1 and EHV-4 mutants, respectively.

### 4.11. Western blotting

Western blot analysis was performed with soluble proteins: 50 μg*/*ml MHC-I, 5 μg*/*ml gD1, and 5 μg*/*ml gD4. Proteins were separated by 12% SDS-PAGE, transferred to a polyvinylidene difluoride (PVDF) membrane (Roth, Karlsruhe, Germany), detected with 1:1000 dilution rabbit anti-His_6_ (Sigma-Aldrich, St Louis, USA) antibody and 1:10000 dilution goat anti-rabbit-HRP antibody (Sigma-Aldrich, St Louis, USA) and visualized by enhanced chemiluminescence (ECL Plus; Amersham).

### 4.12. Virus blocking assays

To block cell surface MHC-I, 1,5×10^5^ ED cells were seeded in 24-well plates. The next day, cells were incubated with 20, 50, 100 or 150 μg/ml recombinant gD1, gD4 or gD4_36-280_ for 1 h on ice. Subsequently cells were infected with either EHV-1 or EHV-4 at MOI = 0.1 and incubated for 1 h at 37°C. To remove un-penetrated viruses, cells were washed with citrate buffer, pH 3, containing 40 mM citric acid, 10 mM potassium chloride and 135 mM sodium chloride, then washed twice with phosphate buffered saline (PBS) and infection allowed to proceed for 24 h (EHV-1) or 48 h (EHV-4). For measurement of fluorescence intensity 10,000 cells were analyzed with a FACSCalibur flow cytometer (BD Biosciences) and the software CytExpert (Beckman Coulter, Krefeld). The experiment was repeated three independent times for each protein.

For plaque reduction assay the same protocol was applied for blocking surface MHC-I with minor changes. Cells were initially incubated with 150 μg*/*ml gD1 or gD4, infected with 100 PFU, and overlaid with 1.5% methylcellulose (Sigma-Aldrich, Taufkirchen, Germany) in Iscove’s Modified Dulbecco’s Medium (IMDM) after citrate treatment and washes with PBS. GFP plaques were counted after 48 h with a Zeiss Axiovert.A1 fluorescent microscope (Carl Zeiss AG, Jena, Germany). The experiment was repeated three independent times for each protein.

### 4.13. Virus growth kinetics

Virus replication was tested using multi-step growth kinetics and plaque areas were obtained as described before (Azab et al., 2010). ED cells were grown to confluency in 24-well plates, infected with an MOI of 0.1 virus and incubated for 1 h at 37°C. Viruses on the cell surface were removed by washing with citrate buffer. After neutralization with IMDM, cells were washed twice with PBS and finally overlaid with 500 μl IMDM. At indicated times after the citrate treatment cells and supernatant were collected separately for EHV-1 and together for EHV-4 and stored at-80°C. Titers were determined by plating dilution series onto ED cells and counting plaque numbers after 2 days under a methylcellulose overlay. All plates were fixed for 10 min with 4% paraformaldehyde, washed with PBS and stained for 10 min with 0.1% crystal violet solution in PBS which was washed away with tab water. Viral titers are expressed as PFU per milliliter from three independent and blinded experiments.

### 4.14. Statistical analysis

For blocking assays, plaque numbers were normalized to infection levels without recombinant proteins. Statistical analysis was done using GraphPad Prism 5 software (San Diego, CA, USA) and one-way ANOVA Bonferroni’s multiple comparison test, * indicates *p ≤* 0.05, ** indicates *p ≤* 0.01, *** indicates *p ≤* 0.001. Statistical analysis was done using an unpaired, one-tailed test. *p* < 0.05 was considered significant.

## Data availability statement

The datasets presented in this study can be found in online repositories. The names of the repository/repositories and accession number(s) can be found at: http://www.wwpdb.org/, 6SQJ; http://www.wwpdb.org/, 6TM8.

## Author contributions

WA, NO, and MW contributed to conception and design of the study. VK solved the crystal structures, performed viral experiments, project administration, validation, visualization, and writing of the original draft. BL analyzed the structure data and solved the crystal structures. SP and GW performed docking experiments including method development, visualization, and writing of sections. CW performed mass spectrometry experiments, analysis, and visualization. ID performed Biacore assays and together with SC analysis and visualization. All authors contributed to the article and approved the submitted version.

## Funding

This work was supported by Deutsche Forschungsgemeinschaft (DFG AZ 97/3–2, www.dfg.de) to WA. The Core Facility BioSupraMol is supported by the Deutsche Forschungsgemeinschaft (DFG project number 213868804) to CW. The funders had no role in study design, data collection and analysis, decision to publish, or preparation of the manuscript.

## Conflict of interest

The authors declare that the research was conducted in the absence of any commercial or financial relationships that could be construed as a potential conflict of interest.

## Publisher’s note

All claims expressed in this article are solely those of the authors and do not necessarily represent those of their affiliated organizations, or those of the publisher, the editors and the reviewers. Any product that may be evaluated in this article, or claim that may be made by its manufacturer, is not guaranteed or endorsed by the publisher.

## References

[ref1] Abdelgawad A. Azab W. Damiani A. M. Baumgartner K. Will H. Osterrieder N.. (2014). Zebra-borne equine herpesvirus type 1 (EHV-1) infection in non-African captive mammals. Vet. Microbiol. 169, 102–106. doi: 10.1016/j.vetmic.2013.12.011, PMID: 24440374

[ref2] Abdelgawad A. Hermes R. Damiani A. Lamglait B. Czirják G. Á. East M.. (2015). Comprehensive serology based on a peptide ELISA to assess the prevalence of closely related equine herpesviruses in zoo and wild animals. PLoS One 10:e0138370. doi: 10.1371/journal.pone.0138370, PMID: 26378452 PMC4574707

[ref3] Adams P. D. Afonine P. V. Bunkóczi G. Chen V. B. Davis I. W. Echols N.. (2010). PHENIX: a comprehensive Python-based system for macromolecular structure solution. Acta Crystallogr. D Biol. Crystallogr. 66, 213–221. doi: 10.1107/S0907444909052925 20124702 PMC2815670

[ref4] Afonine P. V. Grosse-Kunstleve R. W. Echols N. Headd J. J. Moriarty N. W. Mustyakimov M.. (2012). Towards automated crystallographic structure refinement with phenix. Refine. Acta Crystallogr. D Biol. Crystallogr. 68, 352–367. doi: 10.1107/S0907444912001308, PMID: 22505256 PMC3322595

[ref5] Ahn B. C. Zhang Y. O’Callaghan D. J. (2010). The equine herpesvirus-1 (EHV-1) IR3 transcript downregulates expression of the IE gene and the absence of IR3 gene expression alters EHV-1 biological properties and virulence. Virology 402, 327–337. doi: 10.1016/j.virol.2010.03.051, PMID: 20417949 PMC3020145

[ref6] Allen G. (1986). Molecular epizootiology, pathogenesis, and prophylaxis of equine herpesvirus-1 infections. Prog. Vet. Microbiol. Immunol. 2, 78–144. PMID: 2856183

[ref7] Atwood W. J. Norkin L. C. (1989). Class I major histocompatibility proteins as cell surface receptors for simian virus 40. J. Virol. 63, 4474–4477. doi: 10.1128/jvi.63.10.4474-4477.1989, PMID: 2476575 PMC251073

[ref8] Azab W. Gramatica A. Herrmann A. Osterrieder N. (2015). Binding of alphaherpesvirus glycoprotein H to surface α4β1-integrins activates calcium-signaling pathways and induces phosphatidylserine exposure on the plasma membrane. MBio 6, e01552–e01515. doi: 10.1128/mBio.01552-15 26489864 PMC4620472

[ref9] Azab W. Harman R. Miller D. Tallmadge R. Frampton A. R. Jr. Antczak D. F.. (2014). Equid herpesvirus type 4 uses a restricted set of equine major histocompatibility complex class I proteins as entry receptors. J. Gen. Virol. 95, 1554–1563. doi: 10.1099/vir.0.066407-0 24722677

[ref10] Azab W. Kato K. Abdel-Gawad A. Tohya Y. Akashi H. (2011). Equine herpesvirus 4: recent advances using BAC technology. Vet. Microbiol. 150, 1–14. doi: 10.1016/j.vetmic.2011.01.002, PMID: 21292410

[ref11] Azab W. Kato K. Arii J. Tsujimura K. Yamane D. Tohya Y.. (2009). Cloning of the genome of equine herpesvirus 4 strain TH20p as an infectious bacterial artificial chromosome. Arch. Virol. 154, 833–842. doi: 10.1007/s00705-009-0382-0, PMID: 19387789

[ref12] Azab W. Osterrieder K. (2017). Initial Contact: The First Steps in Herpesvirus Entry. Cell Biology of Herpes Viruses Springer, 1–27. 10.1007/978-3-319-53168-7_1 28528437

[ref13] Azab W. Tsujimura K. Maeda K. Kobayashi K. Mohamed Y. M. Kato K.. (2010). Glycoprotein C of equine herpesvirus 4 plays a role in viral binding to cell surface heparan sulfate. Virus Res. 151, 1–9. doi: 10.1016/j.virusres.2010.03.003, PMID: 20236610

[ref14] Azab W. Zajic L. Osterrieder N. (2012). The role of glycoprotein H of equine herpesviruses 1 and 4 (EHV-1 and EHV-4) in cellular host range and integrin binding. Vet. Res. 43:61. doi: 10.1186/1297-9716-43-61, PMID: 22909178 PMC3522555

[ref15] Banks J. L. Beard H. S. Cao Y. Cho A. E. Damm W. Farid R.. (2005). Integrated modeling program, applied chemical theory (IMPACT). J. Comput. Chem. 26, 1752–1780. doi: 10.1002/jcc.20292, PMID: 16211539 PMC2742605

[ref16] Barton G. J. others (1993). ALSCRIPT: a tool to format multiple sequence alignments. Protein Eng. Des. Sel. 6, 37–40. doi: 10.1093/protein/6.1.37, PMID: 8433969

[ref17] Bjorkman P. J. Parham P. (1990). Structure, function, and diversity of class I major histocompatibility complex molecules. Annu. Rev. Biochem. 59, 253–288. doi: 10.1146/annurev.bi.59.070190.001345 2115762

[ref18] Bogan A. A. Thorn K. S. (1998). Anatomy of hot spots in protein interfaces. J. Mol. Biol. 280, 1–9. doi: 10.1006/jmbi.1998.1843, PMID: 9653027

[ref19] Bowers K. J. Chow D. E. Xu H. Dror R. O. Eastwood M. P. Gregersen B. A.. (2006). ACM/IEEE conference on supercomputing. IEEE 2006:43. doi: 10.1145/1188455.1188544

[ref20] Bunkóczi G. Echols N. McCoy A. J. Oeffner R. D. Adams P. D. Read R. J. (2013). Phaser. MRage: automated molecular replacement. Acta Crystallogr. D Biol. Crystallogr. 69, 2276–2286. doi: 10.1107/S0907444913022750, PMID: 24189240 PMC3817702

[ref21] Burrows R. Goodridge D. (1974). In Vivo and in Vitro Studies of Equine Rhinopneumonitis Virus Strains. Equine Infectious Diseases. Basel, Switzerland: Karger Publishers, 306–321.

[ref22] Cairns T. M. Ditto N. T. Atanasiu D. Lou H. Brooks B. D. Saw W. T.. (2019). Surface plasmon resonance reveals direct binding of herpes simplex virus glycoproteins gH/gL to gD and locates a gH/gL binding site on gD. J. Virol. 93, e00289–e00219. doi: 10.1128/JVI.00289-19 31092568 PMC6639271

[ref23] Carfi A. Willis S. H. Whitbeck J. C. Krummenacher C. Cohen G. H. Eisenberg R. J.. (2001). Herpes simplex virus glycoprotein D bound to the human receptor HveA. Mol. Cell 8, 169–179. doi: 10.1016/S1097-2765(01)00298-2, PMID: 11511370

[ref24] Chaudhury S. Berrondo M. Weitzner B. D. Muthu P. Bergman H. Gray J. J. (2011). Benchmarking and analysis of protein docking performance in Rosetta v3. 2. PLoS One 6:e22477. doi: 10.1371/journal.pone.0022477, PMID: 21829626 PMC3149062

[ref25] Chaudhury S. Gray J. J. (2008). Conformer selection and induced fit in flexible backbone protein–protein docking using computational and NMR ensembles. J. Mol. Biol. 381, 1068–1087. doi: 10.1016/j.jmb.2008.05.042, PMID: 18640688 PMC2573042

[ref26] Chen V. B. Arendall W. B. Headd J. J. Keedy D. A. Immormino R. M. Kapral G. J.. (2010). MolProbity: all-atom structure validation for macromolecular crystallography. Acta Crystallogr. D Biol. Crystallogr. 66, 12–21. doi: 10.1107/S0907444909042073, PMID: 20057044 PMC2803126

[ref27] Cole N. L. Grose C. (2003). Membrane fusion mediated by herpesvirus glycoproteins: the paradigm of varicella-zoster virus. Rev. Med. Virol. 13, 207–222. doi: 10.1002/rmv.377, PMID: 12820183

[ref28] Connolly S. A. Whitbeck J. C. Rux A. H. Krummenacher C. Cohen G. H. Eisenberg R. J.. (2001). Glycoprotein D homologs in herpes simplex virus type 1, pseudorabies virus, and bovine herpes virus type 1 bind directly to human HveC (nectin-1) with different affinities. Virology 280, 7–18. doi: 10.1006/viro.2000.0747, PMID: 11162814

[ref29] Consortium U (2019). UniProt: a worldwide hub of protein knowledge. Nucleic Acids Res. 47, D506–D515. doi: 10.1093/nar/gky1049, PMID: 30395287 PMC6323992

[ref30] Csellner H. Walker C. Wellington J. McLure L. Love D. Whalley J. (2000). EHV-1 glycoprotein D (EHV-1 gD) is required for virus entry and cell-cell fusion, and an EHV-1 gD deletion mutant induces a protective immune response in mice. Arch. Virol. 145, 2371–2385. doi: 10.1007/s007050070027, PMID: 11205124

[ref31] David-Watine B. Israël A. Kourilsky P. (1990). The regulation and expression of MHC class I genes. Immunol. Today 11, 286–292. doi: 10.1016/0167-5699(90)90114-O, PMID: 1698378

[ref32] DeLano WL. (2002). PyMOL. “The PyMOL molecular graphics system.” Available at: http://www.pymol.org/

[ref33] Dey P. Bergmann T. Cuellar-Camacho J. L. Ehrmann S. Chowdhury M. S. Zhang M.. (2018). Multivalent flexible Nanogels exhibit broad-Spectrum antiviral activity by blocking virus entry. ACS Nano 12, 6429–6442. doi: 10.1021/acsnano.8b01616, PMID: 29894156

[ref34] Di Giovine P. Settembre E. C. Bhargava A. K. Luftig M. A. Lou H. Cohen G. H.. (2011). Structure of herpes simplex virus glycoprotein D bound to the human receptor nectin-1. PLoS Pathog. 7:e1002277. doi: 10.1371/journal.ppat.1002277, PMID: 21980294 PMC3182920

[ref35] Di Tommaso P. Moretti S. Xenarios I. Orobitg M. Montanyola A. Chang JM. (2011). T-coffee: a web server for the multiple sequence alignment of protein and RNA sequences using structural information and homology extension. Nucleic Acids Res. 39, W13–W17. doi: 10.1093/nar/gkr245 21558174 PMC3125728

[ref36] Ellis S. Martin A. Holmes E. Morrison W. (1995). At least four MHC class I genes are transcribed in the horse: phylogenetic analysis suggests an unusual evolutionary history for the MHC in this species. Int. J. Immunogenet. 22, 249–260. doi: 10.1111/j.1744-313X.1995.tb00239.x, PMID: 8547231

[ref37] Emsley P. Lohkamp B. Scott W. G. Cowtan K. (2010). Features and development of Coot. Acta Crystallogr. D Biol. Crystallogr. 66, 486–501. doi: 10.1107/S0907444910007493, PMID: 20383002 PMC2852313

[ref38] Flowers C. C. Eastman E. M. O’Callaghan D. J. (1991). Sequence analysis of a glycoprotein D gene homolog within the unique short segment of the EHV-1 genome. Virology 180, 175–184. doi: 10.1016/0042-6822(91)90021-3, PMID: 1845821

[ref39] Frampton A. R. Stolz D. B. Uchida H. Goins W. F. Cohen J. B. Glorioso J. C. (2007). Equine herpesvirus 1 enters cells by two different pathways, and infection requires the activation of the cellular kinase ROCK1. J. Virol. 81, 10879–10889. doi: 10.1128/JVI.00504-07, PMID: 17670830 PMC2045510

[ref40] Germain R. N. Margulies D. H. (1993). The biochemistry and cell biology of antigen processing and presentation. Annu. Rev. Immunol. 11, 403–450. doi: 10.1146/annurev.iy.11.040193.002155 8476568

[ref41] Gilcrease M. Z. (2007). Integrin signaling in epithelial cells. Cancer Lett. 247, 1–25. doi: 10.1016/j.canlet.2006.03.031, PMID: 16725254

[ref42] Goehring L. Wagner B. Bigbie R. Hussey S. Rao S. Morley P.. (2010). Control of EHV-1 viremia and nasal shedding by commercial vaccines. Vaccine 28, 5203–5211. doi: 10.1016/j.vaccine.2010.05.065, PMID: 20538091

[ref43] Goodman L. B. Wimer C. Dubovi E. J. Gold C. Wagner B. (2012). Immunological correlates of vaccination and infection for equine herpesvirus 1. Clin. Vaccine Immunol. 19, 235–241. doi: 10.1128/CVI.05522-11, PMID: 22205656 PMC3272919

[ref44] Gowers RJ Linke M Barnoud J Reddy TJE Melo MN Seyler SL. MDAnalysis: A Python Package for the Rapid Analysis of Molecular Dynamics Simulations. Los Alamos National lab.(LANL), Los Alamos, NM (United States); (2019).

[ref45] Gray J. J. Moughon S. Wang C. Schueler-Furman O. Kuhlman B. Rohl C. A.. (2003). Protein–protein docking with simultaneous optimization of rigid-body displacement and side-chain conformations. J. Mol. Biol. 331, 281–299. doi: 10.1016/S0022-2836(03)00670-3 12875852

[ref46] Greenwood A. D. Tsangaras K. Ho S. Y. Szentiks C. A. Nikolin V. M. Ma G.. (2012). A potentially fatal mix of herpes in zoos. Curr. Biol. 22, 1727–1731. doi: 10.1016/j.cub.2012.07.035, PMID: 22902751

[ref47] Handler C. G. Cohen G. H. Eisenberg R. J. (1996). Cross-linking of glycoprotein oligomers during herpes simplex virus type 1 entry. J. Virol. 70, 6076–6082. doi: 10.1128/jvi.70.9.6076-6082.1996, PMID: 8709231 PMC190629

[ref48] Hong S. S. Karayan L. Tournier J. Curiel D. T. Boulanger P. A. (1997). Adenovirus type 5 fiber knob binds to MHC class I α2 domain at the surface of human epithelial and B lymphoblastoid cells. EMBO J. 16, 2294–2306. doi: 10.1093/emboj/16.9.2294, PMID: 9171344 PMC1169831

[ref49] Humphrey W Dalke A Schulten K, others. VMD: visual molecular dynamics. J. Mol. Graph. (1996); 14: 33–38, doi: 10.1016/0263-7855(96)00018-5 8744570

[ref50] JBBM Weitzner Kilambi K Thottungal R Chaudhury S Wang C Gray. (2017). Docking Protocol (Rosetta Dock).

[ref51] Kabsch W. (2010). XDS. Acta Crystallogr. D Biol. Crystallogr. 66, 125–132. doi: 10.1107/S0907444909047337 20124692 PMC2815665

[ref52] Kabsch W. Sander C. (1983). DSSP: definition of secondary structure of proteins given a set of 3D coordinates. Biopolymers 22, 2577–2637. doi: 10.1002/bip.360221211 6667333

[ref53] Kaminski G. A. Friesner R. A. Tirado-Rives J. Jorgensen W. L. (2001). Evaluation and reparametrization of the OPLS-AA force field for proteins via comparison with accurate quantum chemical calculations on peptides. J. Phys. Chem. B 105, 6474–6487. doi: 10.1021/jp003919d

[ref54] Krummenacher C. Carfí A. Eisenberg R. J. Cohen G. H. (2013). Entry of Herpesviruses into Cells: The Enigma Variations. Viral Entry into Host Cells. Cham, Switzerland: Springer, 178–195. 10.1007/978-1-4614-7651-1_10 23884592

[ref55] Krummenacher C. Rux A. H. Whitbeck J. C. Ponce-de-Leon M. Lou H. Baribaud I.. (1999). The first immunoglobulin-like domain of HveC is sufficient to bind herpes simplex virus gD with full affinity, while the third domain is involved in oligomerization of HveC. J. Virol. 73, 8127–8137. doi: 10.1128/JVI.73.10.8127-8137.1999, PMID: 10482562 PMC112829

[ref56] Krummenacher C. Supekar V. M. Whitbeck J. C. Lazear E. Connolly S. A. Eisenberg R. J.. (2005). Structure of unliganded HSV gD reveals a mechanism for receptor-mediated activation of virus entry. EMBO J. 24, 4144–4153. doi: 10.1038/sj.emboj.7600875, PMID: 16292345 PMC1356314

[ref57] Kurtz B. M. Singletary L. B. Kelly S. D. Frampton A. R. (2010). *Equus caballus* major histocompatibility complex class I is an entry receptor for equine herpesvirus type 1. J. Virol. 84, 9027–9034. doi: 10.1128/JVI.00287-10, PMID: 20610718 PMC2937649

[ref58] Kydd J. H. Townsend H. G. Hannant D. (2006). The equine immune response to equine herpesvirus-1: the virus and its vaccines. Vet. Immunol. Immunopathol. 111, 15–30. doi: 10.1016/j.vetimm.2006.01.005 16476492

[ref59] Labute P. (2008). The generalized born/volume integral implicit solvent model: estimation of the free energy of hydration using London dispersion instead of atomic surface area. J. Comput. Chem. 29, 1693–1698. doi: 10.1002/jcc.20933, PMID: 18307169

[ref60] Labute P. (2009). Protonate 3D: assignment of ionization states and hydrogen coordinates to macromolecular structures. Proteins: Struct., Funct., Bioinf. 75, 187–205. doi: 10.1002/prot.22234, PMID: 18814299 PMC3056144

[ref61] Lazear E. Carfi A. Whitbeck J. C. Cairns T. M. Krummenacher C. Cohen G. H.. (2008). Engineered disulfide bonds in herpes simplex virus type 1 gD separate receptor binding from fusion initiation and viral entry. J. Virol. 82, 700–709. doi: 10.1128/JVI.02192-07, PMID: 18032483 PMC2224591

[ref62] Li A. Lu G. Qi J. Wu L. Tian K. Luo T.. (2017). Structural basis of nectin-1 recognition by pseudorabies virus glycoprotein D. PLoS Pathog. 13:e1006314. doi: 10.1371/journal.ppat.1006314, PMID: 28542478 PMC5453625

[ref63] Liu Q. Li J. (2010). Protein binding hot spots and the residue-residue pairing preference: a water exclusion perspective. BMC Bioinf. 11:244. doi: 10.1186/1471-2105-11-244, PMID: 20462403 PMC2882391

[ref64] Lu G. Zhang N. Qi J. Li Y. Chen Z. Zheng C.. (2014). Crystal structure of herpes simplex virus 2 gD bound to nectin-1 reveals a conserved mode of receptor recognition. J. Virol. 88, 13678–13688. doi: 10.1128/JVI.01906-14, PMID: 25231300 PMC4248990

[ref65] Manoj S. Jogger C. R. Myscofski D. Yoon M. Spear P. G. (2004). Mutations in herpes simplex virus glycoprotein D that prevent cell entry via nectins and alter cell tropism. Proc. Natl. Acad. Sci. 101, 12414–12421. doi: 10.1073/pnas.0404211101, PMID: 15273289 PMC515077

[ref66] Michaud-Agrawal N. Denning E. J. Woolf T. B. Beckstein O. (2011). MDAnalysis: a toolkit for the analysis of molecular dynamics simulations. J. Comput. Chem. 32, 2319–2327. doi: 10.1002/jcc.21787, PMID: 21500218 PMC3144279

[ref67] Mullen M. M. Haan K. M. Longnecker R. Jardetzky T. S. (2002). Structure of the Epstein-Barr virus gp 42 protein bound to the MHC class II receptor HLA-DR1. Mol. Cell 9, 375–385. doi: 10.1016/S1097-2765(02)00465-3, PMID: 11864610

[ref68] Norkin L. C. (1999). Simian virus 40 infection via MHC class I molecules and caveolae. Immunol. Rev. 168, 13–22. doi: 10.1111/j.1600-065X.1999.tb01279.x, PMID: 10399061

[ref69] Osterrieder N. (1999). Construction and characterization of an equine herpesvirus 1 glycoprotein C negative mutant. Virus Res. 59, 165–177. doi: 10.1016/S0168-1702(98)00134-8, PMID: 10082388

[ref70] Osterrieder N. Van de Walle G. R. (2010). Pathogenic potential of equine alphaherpesviruses: the importance of the mononuclear cell compartment in disease outcome. Vet. Microbiol. 143, 21–28. doi: 10.1016/j.vetmic.2010.02.010, PMID: 20202764

[ref71] Patel J. Heldens J. (2005). Equine herpesviruses 1 (EHV-1) and 4 (EHV-4)–epidemiology, disease and immunoprophylaxis: a brief review. Vet. J. 170, 14–23. doi: 10.1016/j.tvjl.2004.04.018, PMID: 15993786

[ref72] Prilusky J. Felder C. E. Zeev-Ben-Mordehai T. Rydberg E. H. Man O. Beckmann J. S.. (2005). FoldIndex©: a simple tool to predict whether a given protein sequence is intrinsically unfolded. Bioinformatics 21, 3435–3438. doi: 10.1093/bioinformatics/bti537, PMID: 15955783

[ref73] Ramachandran G. N. (1963). Stereochemistry of polypeptide chain configurations. J. Mol. Biol. 7, 95–99. doi: 10.1016/S0022-2836(63)80023-6 13990617

[ref74] Robert X. Gouet P. (2014). Deciphering key features in protein structures with the new ENDscript server. Nucleic Acids Res. 42, W320–W324. doi: 10.1093/nar/gku316, PMID: 24753421 PMC4086106

[ref75] Rudolph J. O’Callaghan D. Osterrieder N. (2002). Cloning of the genomes of equine herpesvirus type 1 (EHV-1) strains KyA and RacL11 as bacterial artificial chromosomes (BAC). J. Veterinary Med. Ser. B 49, 31–36. doi: 10.1046/j.1439-0450.2002.00534.x 11911590

[ref76] Rux A. H. Willis S. H. Nicola A. V. Hou W. Peng C. Lou H.. (1998). Functional region IV of glycoprotein D from herpes simplex virus modulates glycoprotein binding to the herpesvirus entry mediator. J. Virol. 72, 7091–7098. doi: 10.1128/JVI.72.9.7091-7098.1998, PMID: 9696802 PMC109930

[ref77] Santos K. F. Jovin S. M. Weber G. Pena V. Lührmann R. Wahl M. C. (2012). Structural basis for functional cooperation between tandem helicase cassettes in Brr2-mediated remodeling of the spliceosome. Proc. Natl. Acad. Sci. 109, 17418–17423. doi: 10.1073/pnas.1208098109, PMID: 23045696 PMC3491510

[ref78] Sasaki M. Hasebe R. Makino Y. Suzuki T. Fukushi H. Okamoto M.. (2011). Equine major histocompatibility complex class I molecules act as entry receptors that bind to equine herpesvirus-1 glycoprotein D. Genes Cells 16, 343–357. doi: 10.1111/j.1365-2443.2011.01491.x, PMID: 21306483 PMC3118799

[ref79] Sasaki M. Kim E. Igarashi M. Ito K. Hasebe R. Fukushi H.. (2011). Single amino acid residue in the A2 domain of major histocompatibility complex class I is involved in the efficiency of equine herpesvirus-1 entry. J. Biol. Chem. 286, 39370–39378. doi: 10.1074/jbc.M111.251751, PMID: 21949188 PMC3234761

[ref80] Schasfoort R. B. (2017). Handbook of Surface Plasmon Resonance. Piccadilly, London: Royal Society of Chemistry.

[ref81] Scheurer M. Rodenkirch P. Siggel M. Bernardi R. C. Schulten K. Tajkhorshid E.. (2018). PyContact: rapid, customizable, and visual analysis of noncovalent interactions in MD simulations. Biophys. J. 114, 577–583. doi: 10.1016/j.bpj.2017.12.003, PMID: 29414703 PMC5985026

[ref82] Sievers F. Wilm A. Dineen D. Gibson T. J. Karplus K. Li W.. (2011). Fast, scalable generation of high-quality protein multiple sequence alignments using Clustal omega. Mol. Syst. Biol. 7:539. doi: 10.1038/msb.2011.75, PMID: 21988835 PMC3261699

[ref83] Sircar A. Chaudhury S. Kilambi K. P. Berrondo M. Gray J. J. (2010). A generalized approach to sampling backbone conformations with Rosetta dock for CAPRI rounds 13–19. Proteins: Struct., Funct., Bioinf. 78, 3115–3123. doi: 10.1002/prot.22765, PMID: 20535822 PMC2952725

[ref84] Spear P. G. Longnecker R. (2003). Herpesvirus entry: an update. J. Virol. 77, 10179–10185. doi: 10.1128/JVI.77.19.10179-10185.2003, PMID: 12970403 PMC228481

[ref85] Studdert M. Blackney M. (1979). Equine herpesviruses: on the differentiation of respiratory from foetal strains of type 1. Aust. Vet. J. 55, 488–492. doi: 10.1111/j.1751-0813.1979.tb00377.x, PMID: 231960

[ref86] Tallmadge R. L. Campbell J. A. Miller D. C. Antczak D. F. (2010). Analysis of MHC class I genes across horse MHC haplotypes. Immunogenetics 62, 159–172. doi: 10.1007/s00251-009-0420-9, PMID: 20099063 PMC2872545

[ref87] Tarazona M. P. Saiz E. (2003). Combination of SEC/MALS experimental procedures and theoretical analysis for studying the solution properties of macromolecules. J. Biochem. Biophys. Methods 56, 95–116. doi: 10.1016/S0165-022X(03)00075-7, PMID: 12834971

[ref88] Tischer K. B. von Einem J. Kaufer B. Osterrieder N. (2006). Two-step red-mediated recombination for versatile high-efficiency markerless DNA manipulation in *Escherichia coli*. BioTechniques 40, 191–197. doi: 10.2144/000112096, PMID: 16526409

[ref89] Toukan K. Rahman A. (1985). Molecular-dynamics study of atomic motions in water. Phys. Rev. B 31, 2643–2648. doi: 10.1103/PhysRevB.31.2643 9936106

[ref90] Triantafilou K. Fradelizi D. Wilson K. Triantafilou M. (2002). GRP78, a coreceptor for coxsackievirus A9, interacts with major histocompatibility complex class I molecules which mediate virus internalization. J. Virol. 76, 633–643. doi: 10.1128/JVI.76.2.633-643.2002, PMID: 11752154 PMC136810

[ref91] van Rossum G. (ed.) (1995). Python Tutorial, Technical Report CS-R9526, Centrum Voor Wiskunde en Informatica (CWI), The Netherlands: Centrum voor Wiskunde en Informatica.

[ref92] Whitbeck J. C. Muggeridge M. I. Rux A. H. Hou W. Krummenacher C. Lou H.. (1999). The major neutralizing antigenic site on herpes simplex virus glycoprotein D overlaps a receptor-binding domain. J. Virol. 73, 9879–9890. doi: 10.1128/JVI.73.12.9879-9890.1999, PMID: 10559300 PMC113037

[ref93] Williams C. J. Headd J. J. Moriarty N. W. Prisant M. G. Videau L. L. Deis L. N.. (2018). MolProbity: more and better reference data for improved all-atom structure validation. Protein Sci. 27, 293–315. doi: 10.1002/pro.3330, PMID: 29067766 PMC5734394

[ref94] Willis S. H. Rux A. H. Peng C. Whitbeck J. C. Nicola A. V. Lou H.. (1998). Examination of the kinetics of herpes simplex virus glycoprotein D binding to the herpesvirus entry mediator, using surface plasmon resonance. J. Virol. 72, 5937–5947. doi: 10.1128/JVI.72.7.5937-5947.1998, PMID: 9621056 PMC110398

[ref95] Yang H. Guranovic V. Dutta S. Feng Z. Berman H. M. Westbrook J. D. (2004). Automated and accurate deposition of structures solved by X-ray diffraction to the protein data Bank. Acta Crystallogr. D Biol. Crystallogr. 60, 1833–1839. doi: 10.1107/S0907444904019419 15388930

[ref96] Yao S. Liu J. Qi J. Chen R. Zhang N. Liu Y.. (2016). Structural illumination of equine MHC class I molecules highlights unconventional epitope presentation manner that is evolved in equine leukocyte antigen alleles. J. Immunol. 196, 1943–1954. doi: 10.4049/jimmunol.1501352, PMID: 26764037

[ref97] Yue D. Chen Z. Yang F. Ye F. Lin S. He B.. (2020). Crystal structure of bovine herpesvirus 1 glycoprotein D bound to nectin-1 reveals the basis for its low-affinity binding to the receptor. Sci. Adv. 6:5147. doi: 10.1126/sciadv.aba5147 PMC7220272 32426511

[ref98] Zhang N. Yan J. Lu G. Guo Z. Fan Z. Wang J.. (2011). Binding of herpes simplex virus glycoprotein D to nectin-1 exploits host cell adhesion. Nat. Commun. 2:577. doi: 10.1038/ncomms1571, PMID: 22146396

