# Supplementary material for: Crystal structures of glycoprotein D of equine alphaherpesviruses reveal potential binding sites to the entry receptor MHC-I
Source: Front Microbiol. 2023 May 11;14:1197120. doi: 10.3389/fmicb.2023.1197120 (PMC10213783; doi:10.3389/fmicb.2023.1197120)
Supplement: Supplementary file 1 [file Data_Sheet_1.pdf]

Supplementary material

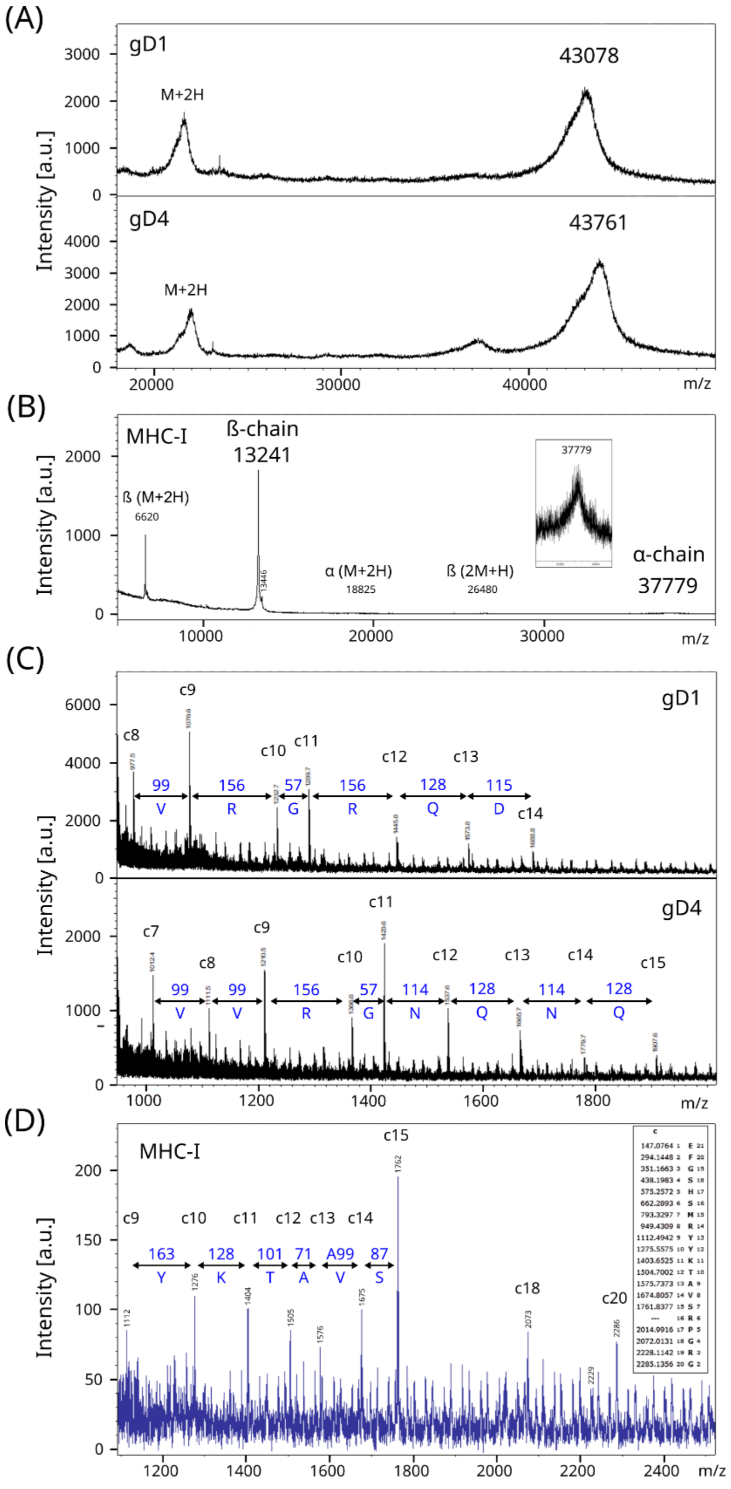

Figure S 1: Mass spectrometric analysis of gD1 and gD4.
(A) Intact protein mass analysis of recombinant gD1 (top) and gD4 (bottom) including N-terminal residues (EF: glutamic acid and phenylalanine) from *Eco*RI restriction site, TEV cleavage site and His$_6$-tag on sinapinic acid (SA) matrix. (B) Intact protein mass analysis of recombinant MHC-I complex comprised of β2m and MHC-I-α-chain (insert zoom) including the same additional residues as gD1 and gD4. (C) In-source decay (ISD) spectra of recombinant gD1 (top), gD4 (bottom), and (D) MHC-I to ascertain the correct N-termini, insert: Theoretical c-ion series including the N-terminal EF extension.

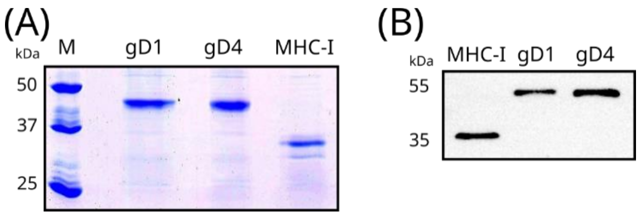

Figure S 2: SDS-PAGE and western blot of MHC-I, gD1, and gD4.
(A) Coomassie stained SDS-PAGE on 12% gel. For MHC-I, only the α-chain is visible in (A) and (B). M = marker. (B) Western blot: MHC-I (50 µg/ml), gD1 (5 µg/ml), and gD4 (5 µg/ml) detected with 1:1000 rabbit anti-His$_6$ antibody and 1:10000 goat anti-rabbit-HRP as secondary antibody.

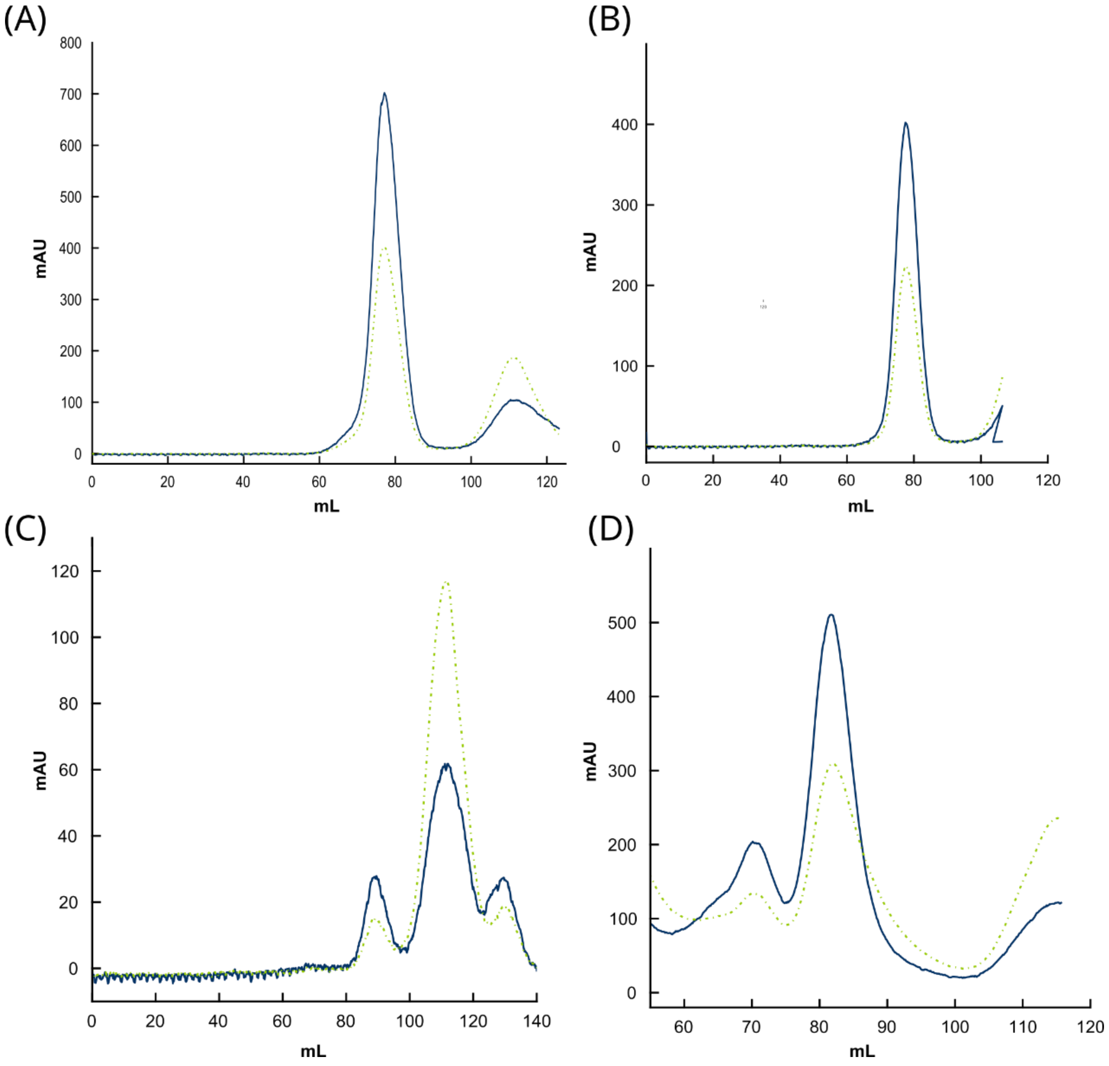

Figure S 3: Protein purification by size exclusion chromatography (SEC).
Representative SEC curves of concentrated (A) gD1, (B) gD4, (C) gD4$_{36\text{-}280}$, and (D) MHC-I run on Superdex 200 16/600 after purification through immobilized metal ion affinity chromatography (IMAC). Solid curves show UV absorbance at 280 nm and the dotted curves at 260 nm.

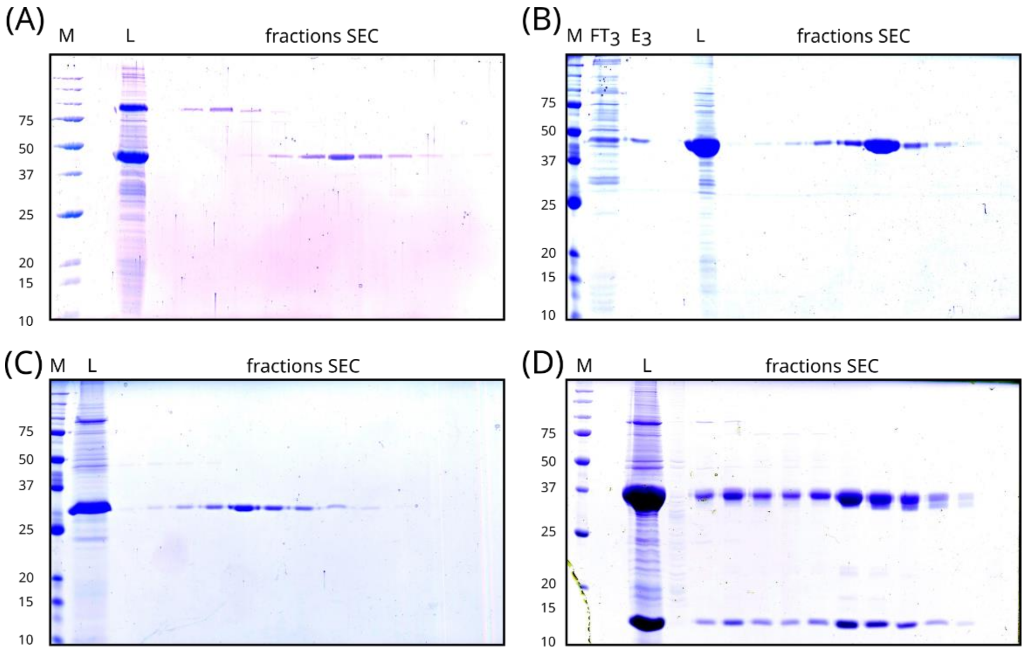

Figure S 4: SDS-PAGE of protein purification by size exclusion chromatography (SEC). Representative size exclusion chromatography (SEC) fractions of proteins produced in insect cells on Coomassie stained 12% sodium dodecyl sulfate (SDS) gels. (A) gD1 (approximately 43 kDa), (B) gD4 (approximately 43 kDa), (C) $gD4_{36-280}$ (approximately 30 kDa), and (D) MHC-I (comprised of α-chain with an approximate size of 38 kDa and β2m (with the linker and peptide) with an approximate size of 13 kDa). M = marker, FT = flow through affinity chromatography, E = elution affinity chromatography, L = loaded on SEC column.

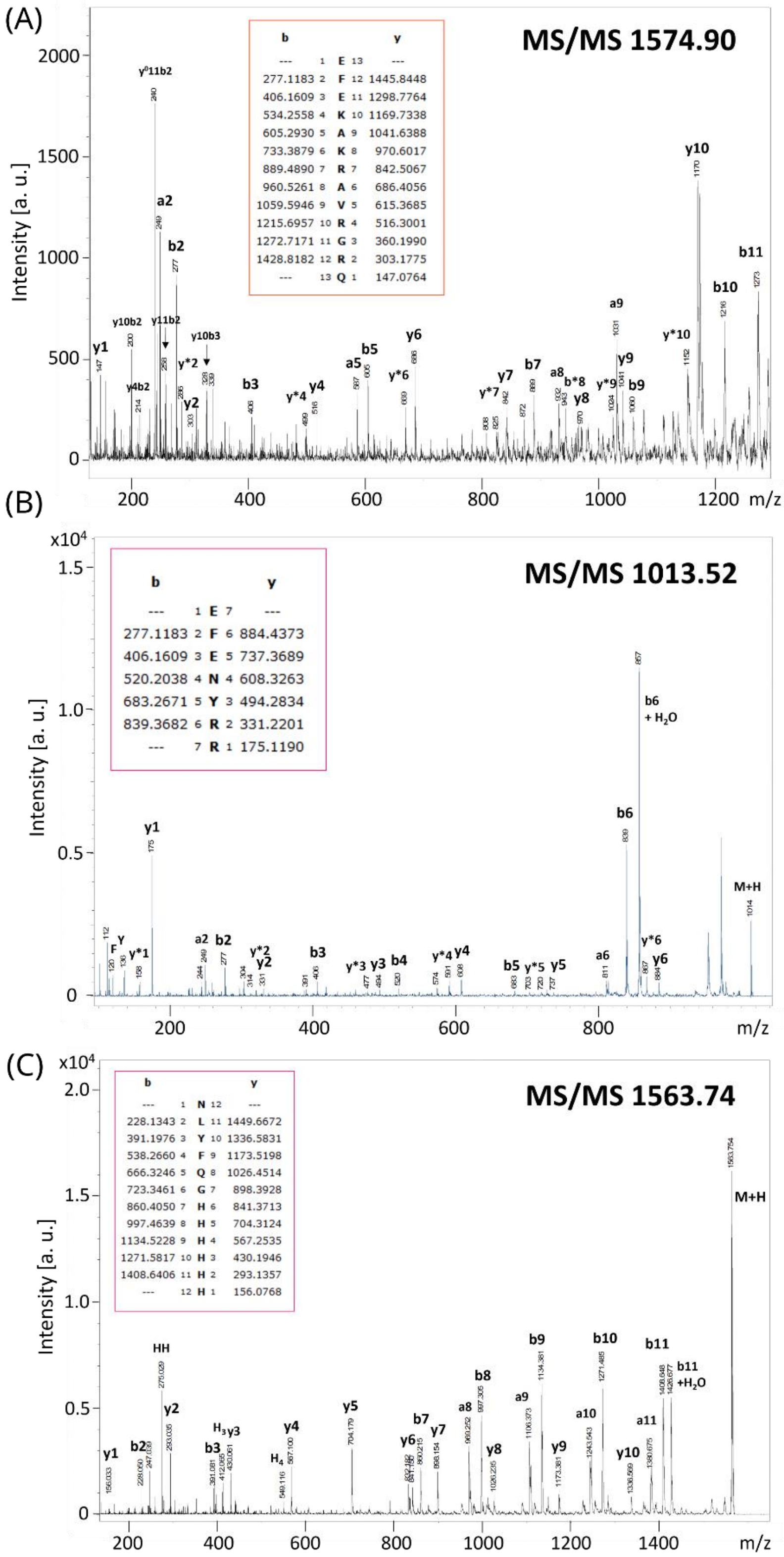

Figure S 5: Tandem mass spectrometry (MS/MS) of in-gel digested gD1 and gD4. Exemplary spectra are shown which confirm the identity of the analyzed proteins and the integrity of their termini. The inserts display the theoretical b and y fragments ions.

(A) N-terminal peptide of gD1 generated by cleavage with Asp-N endoproteinase (M+H= 1574.90, pos. 001-013, sequence EFEKAKRAVRGRQ.D); (B) N-terminal peptide of gD4 obtained by trypsin cleavage (M+H=1013.52, pos. 001-007, sequence EFENYRR); (C) C-terminal peptide of gD4 including the His$_6$-tag, generated by Glu-C endoproteinase (M+H=1563.74, pos. 323-334, sequence ENLYFQG-H$_6$).

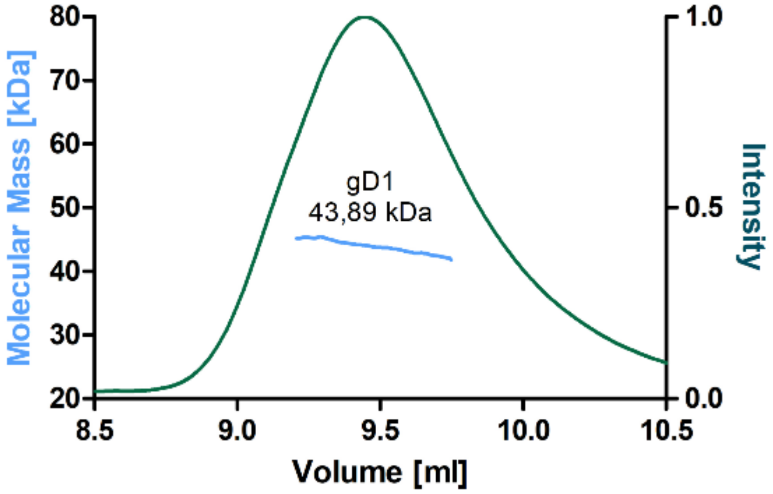

Figure S 6: Size exclusion chromatography (SEC) combined with multi-angle static light scattering (MALS) analysis of gD1.

The gD1 crystal structure consists of a homodimer with two ions interpreted as magnesium originating from the crystallization solution, trapped between them. The ionic interaction together with a high Complex Formation Significance Score of 0.765 (PDB Proteins, Interfaces, Structures and Assemblies (PISA) server www.ebi.ac.uk/pdbe/pisa/) suggested that gD1 might form a dimer on the virus envelope as has been proposed for HSV-1 gD [26]. To evaluate whether recombinant gD of EHV-1 has a homodimeric and/or monomeric form in solution, molecular mass calculation based on SEC-MALS analysis was performed for gD1. Green curve represents the normalized refractive index trace (intensity, right y-axis) for gD1 eluted from a Superdex 200 10/300 column. Blue line under the peak corresponds to the averaged molecular mass distribution (left y axis) across the peak. Exclusively the monomeric form with an approximate molecular weight of 44 kDa was detected. It can be concluded that gD1 is a monomer in solution.

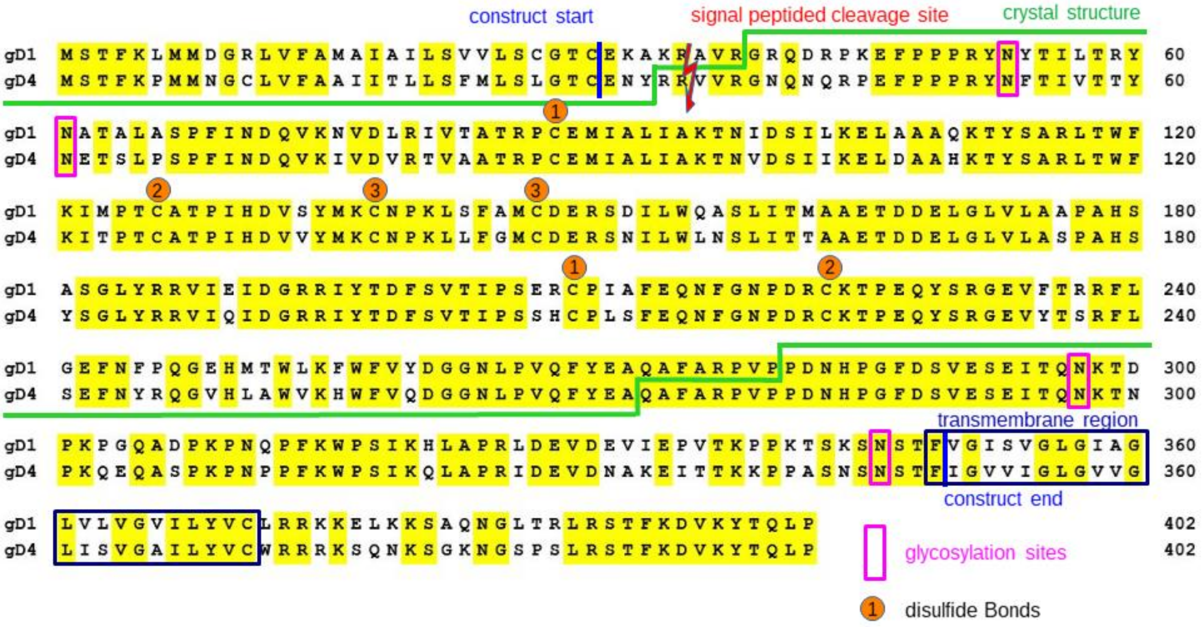

Figure S 7: Sequence alignment of EHV-1 and EHV-4 gD. Yellow marked residues are identical. Expression construct region for gD1 and gD4 is indicated with blue lines, green marked sequences are seen in the crystal structures, disulfide bonds are numbered and marked with orange circles, glycosylation sites are marked with pink boxes, signal peptide cleavage site is indicated by a red bolt, and the transmembrane region is marked blue.

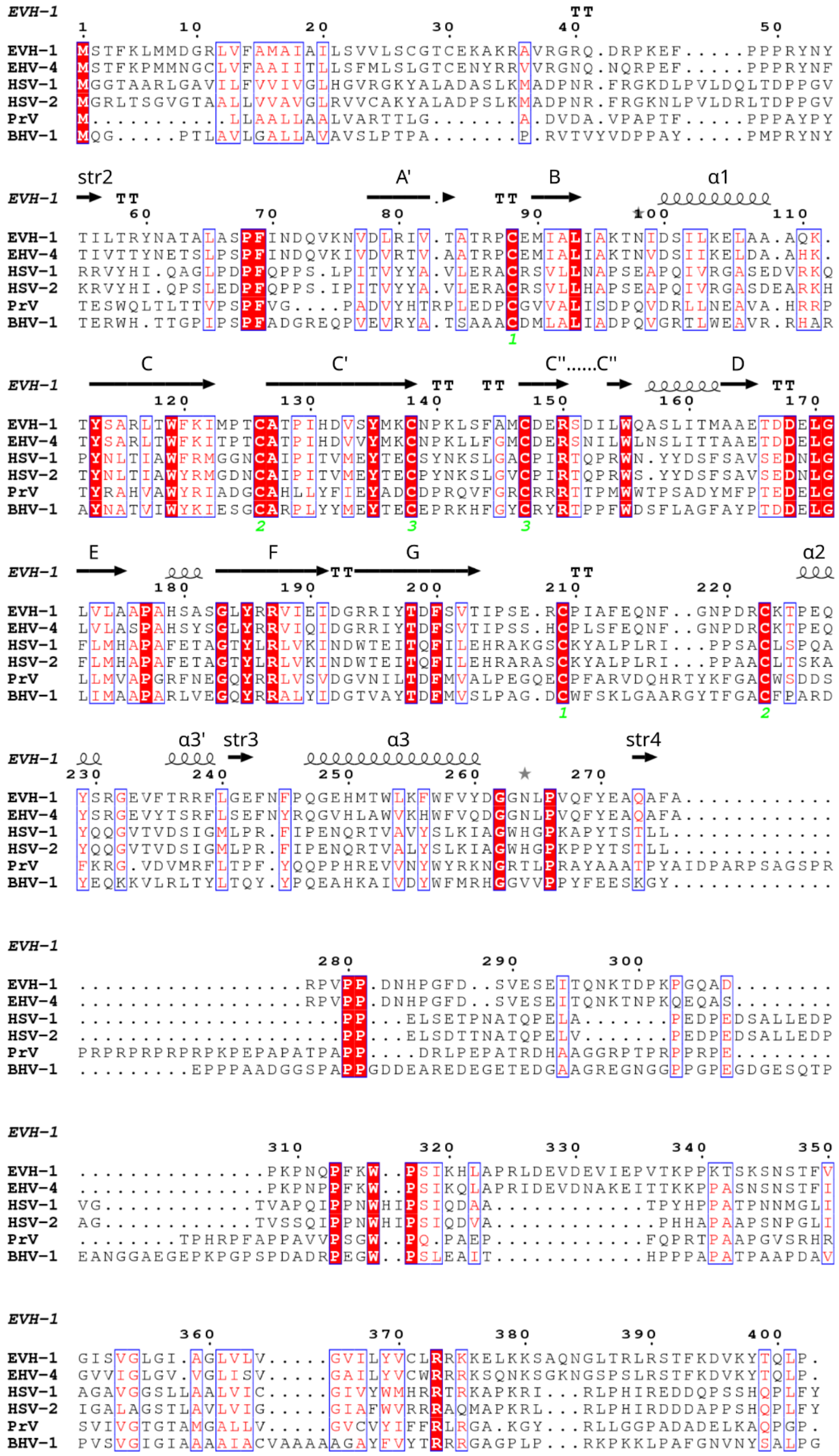

Figure S 8: Sequence alignment of EHV-1, EHV-4, PrV, HSV-1, HSV-2, and BHV-1. Sequence alignment by using structural information was done with the web tool Tcoffee Expresso [97] and the output was modified with ESPript [98]. Secondary structure assignment is according to ESPript. The reference sequence and secondary structure assignment are EHV-1. Sheets are indicated as arrows, helices as cureld lines, disulfide bonds are labeled with green numbers, glycosylation sites as grey stars,. Labels of secondary structures correspond to the naming scheme presented by Li et al. [29]

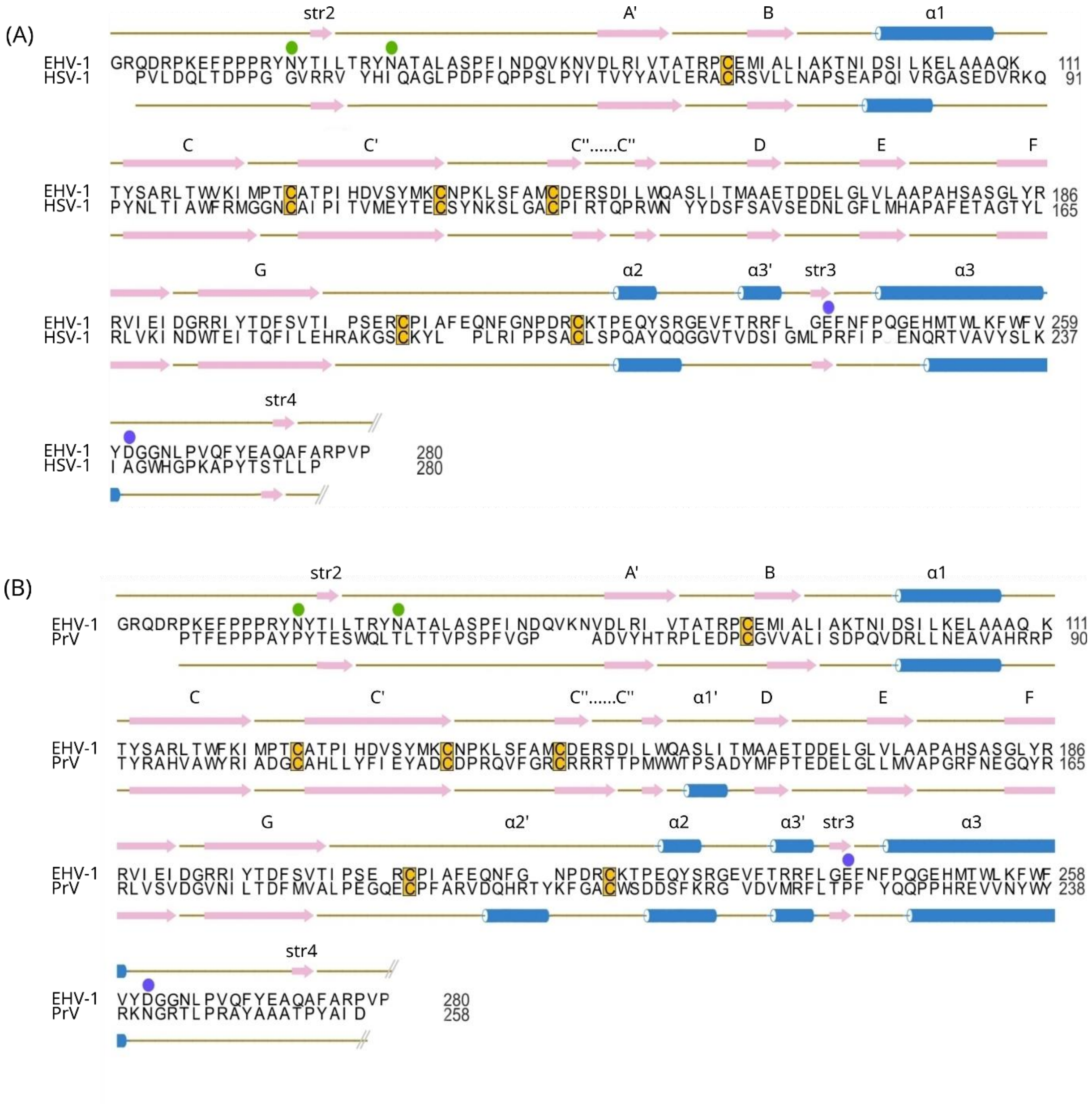

Figure S 9: Sequence alignment based on secondary structure of EHV-1 with HSV-1 and PrV.

Sequence alignment based on secondary structures of gD1 with (A) HSV-1 (PDB ID 2C3A) and (B) PrV (PDB ID 5X5V) gD according to dssp [73]. Sheets are indicated as pink arrows, helices as blue cylinder, disulfide bonds as yellow boxes, glycosylation sites in gD1 as green dots, and magnesium coordinating residues in gD1 as purple dots. Labels correspond to the naming scheme presented by Li et al. [29].

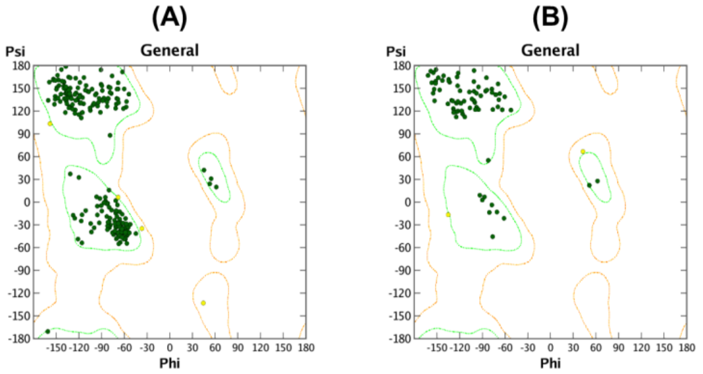

Figure S 10: Ramachandran plots for modeled MHC-I.
Ramachandran plots for (A) MHC-I (gene 3.1) and (B) equine β2m. Symbol code: green point- residue with favorable geometry, yellow point- residue with allowed geometry.

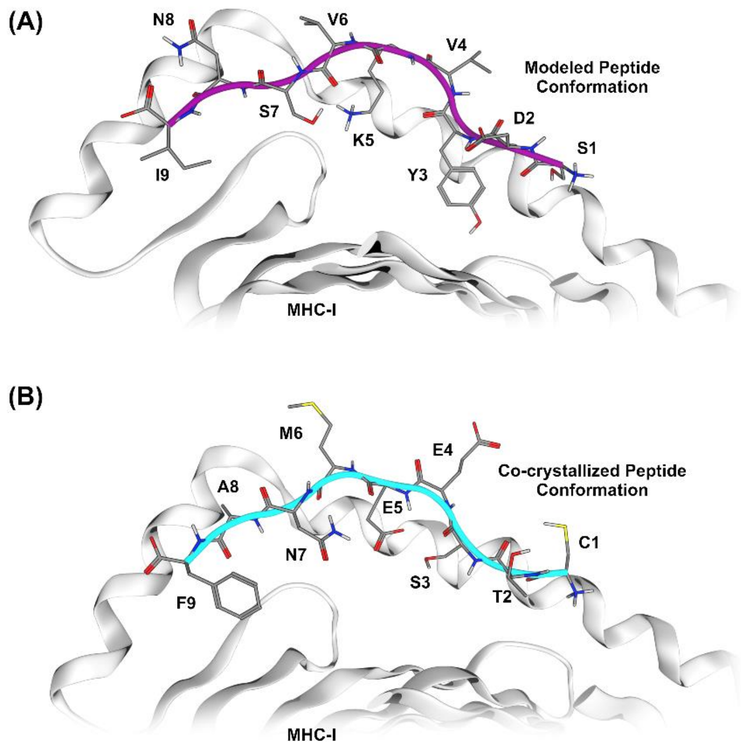

Figure S 11: Peptide conformation in MHC-I homology model. (A) Peptide conformation (SDYVKVSNI, purple ribbon) hypothesis in the MHC-I gene 3.1 (grey ribbon); (B) Template peptide conformation (CTSEEMNAF, cyan ribbon) co-crystallized in the MHC-I genotype EqcaN*00602 (grey ribbon, PDB-ID: 4ZUU (Yao et al., 2016)). For optimized view, the α1 helix of MHC-I is hidden.

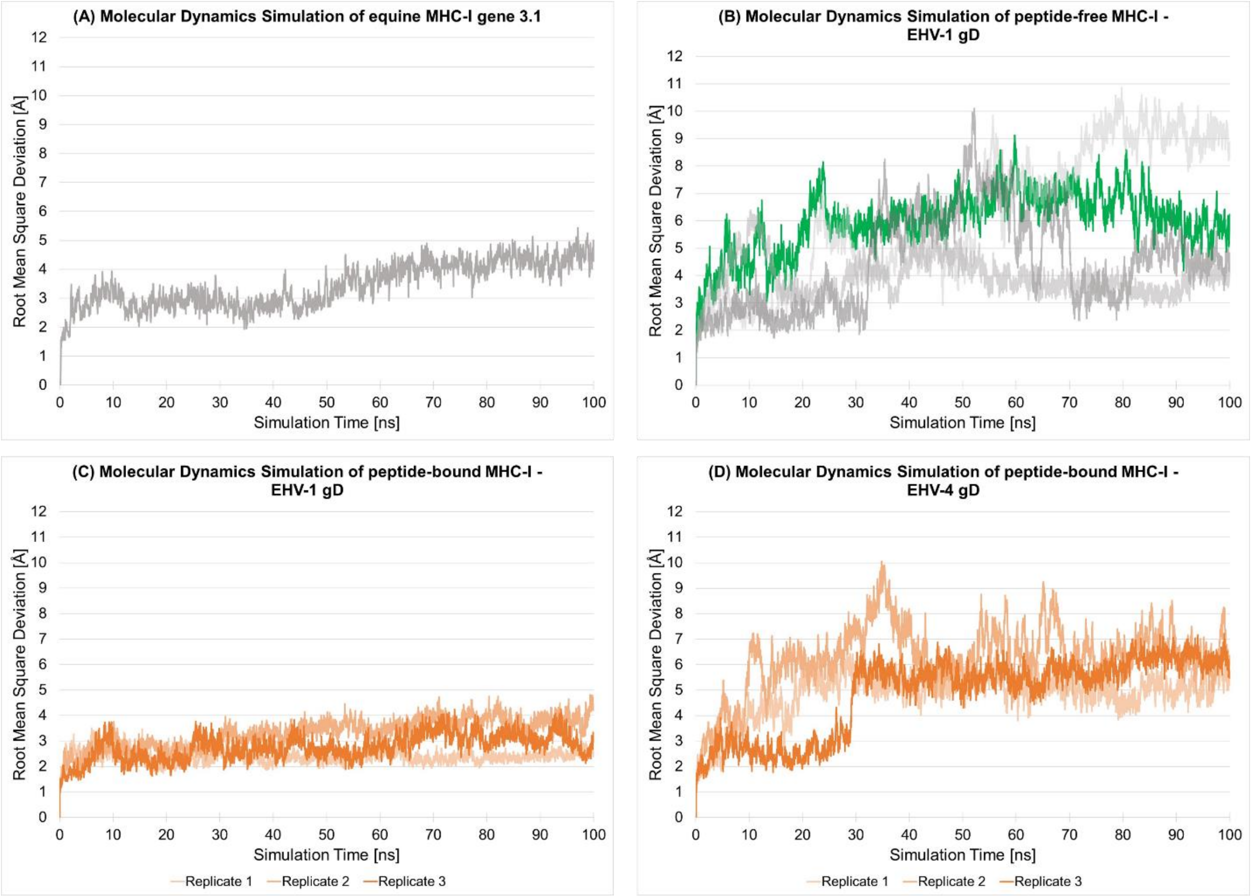

Figure S 12: Root-mean-square-deviation plots from molecular dynamics simulations. To test whether additional bias emerging from peptide modeling influenced docking experiments, two docking rounds were performed. First, gD1 was docked to peptide-free MHC-I. Second, gD1 was docked to MHC-I 3.1 homology model containing the peptide SDYVKVSNI to check if docking provides comparable PPIs. Shown are backbone root-mean-square-deviation (RMSD) plots from molecular dynamics (MD) simulations of: (A) the homology model of equine MHC-I gene 3.1 and equine β2m (single MD simulation), (B) four selected peptide-free EHV-1 gD – MHC-I docking poses (single MD simulation, each; green-analyzed most stable complex), and the final peptide-bound EHV-1 (C) / EHV-4 (D) – MHC-I docking poses (three MD replicates, each).

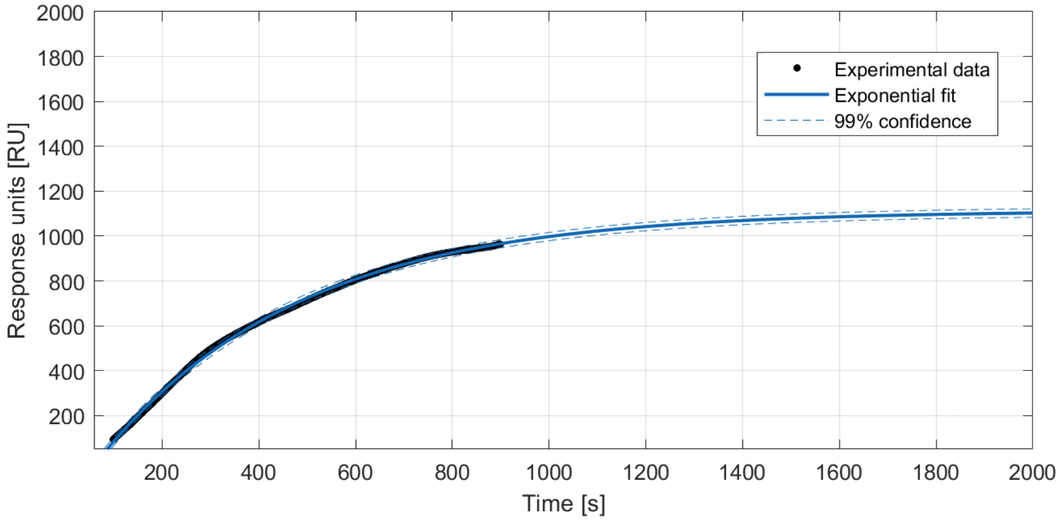

Figure S 13: Equilibrium extrapolation for SPR binding phase curves. For the cases in which the obtained binding curves (see e.g. Figure 3 A-C) did not reach a clearly discernible equilibrium plateau, we extrapolated the equilibrium response by fitting a simple exponential growth model to the SPR sensorgram. The model used was $RU = a * \left(1 - exp\left(-\frac{time-b}{c}\right)\right)$, where a, b and c are free parameters. In this figure, the procedure is shown for a single experiment in the context of gD1 at 5.6 µM concentration, as an example.

Table S 1: Predicted and measured molecular mass in Da of recombinant gD1, gD4 (aa 31-349), and equine MHC-I 3.1 α and β2m region with the uncleaved TEV site and His$_6$-tag. Theoretical masses were calculated using https://web.expasy.org/peptide_mass/ and the actual mass determined by matrix-assisted laser desorption ionization-time of flight mass spectrometry (MALDI-TOF-MS). Post-translational modifications like glycosylations account for the discrepancies between the predictions and measurements. NA: not applicable.

To this point, molecular masses of gD1 and gD4 were determined only by SDS-PAGE and Western blotting. However, these techniques are known to often lead to an overestimation of the molecular mass [30]. Here we employed matrix-assisted laser desorption ionization-time of flight mass spectrometry (MALDI-TOF-MS) to analyze diluted recombinant protein. The proteins gD1, gD4 and the MHC-I α-chain contain a Tobacco Etch Virus (TEV) cleavage site and a His$_6$-tag (ENLYFQG-H$_6$) which contribute approximately 1675 Da to the molecular weight of the molecules (calculated with https://web.expasy.org/peptide_mass). Additionally, the residues EF (approximately 300 Da) originating from the Eco-RI restriction site, were detected by in-source-decay (ISD) in the recombinant proteins. A size of approximately 43078 Da for gD1, 43761 Da for gD4, 37779 Da for the α-chain of MHC-I and 13241 Da for β2m with its linker and attached peptide was determined (
Figure S **1**). Excluding the molecular weight of the TEV cleavage site and the His$_6$-tag (1675 Da), this translates into a molecular weight of 41403 and 42086 Da for soluble gD1 and gD4, respectively and implies an approximate molecular weight of 49345 Da for the recombinant MHC-I molecule consisting of α-chain (36100 Da) and β2m with linker and peptide (13240 Da), and 48500 Da without the linker. The difference between predicted and observed molecular masses is due to post translational modifications (PTMs) such as glycosylations which contribute approximately 4000-5000 Da. Further analysis of recombinant gD1, gD4, and MHC-I by ISD and tandem mass spectrometry (MS/MS) of in-gel digested Coomassie-stained proteins confirmed protein identities and presence of the correct N- and C-termini of gD1 and gD4 and N-terminus of MHC-I α-chain (Figure S 5).

**Molecule**	**predicted**	**measured**	**without TEV and His6**
gD1	38216	43078	41403
gD4	38252	43761	42086
MHC-I α 1-3	33399	37779	36104
β2m (+linker and peptide)	13243	13241	NA

Table S 2: **Primers used for sequencing and construction of protein production plasmids, mutant and revertant viruses.**

Primer	Name	Sequence
VK6	Ph+gp64SP fwd	attataatcgattcgcgacctactcc
VK7	Ph+gp64SP rev	tatatagaattccgcaaaggcagaat
VK8	gD1 for	atattaccatggagaaagccaagcgtgcg
VK10	gD4 for	atatatccatggaaaattacaggcgtgtggttcg
VK18	Ph+gp64SP	atcgattcgcgacctactccggaatattaatagatcatggagataattaaaatga taaccatctcgcaaataaataagtattttactgttttcgtaacagttttgtaata aaaaaacctataaatattccggattattcataccgtcccaccatcgggcgcatgg taagcgctattgttttatatgtgcttttggcggcggcggcgcattctgcctttgc ggaattc
VK35	alpha1-3 for	atatatgaattcggtagccactcaatg
VK38	β2m rev	tatataagtactttagtggtggtggtggtggtgcaggtcacggtcccactta
VK50	EcoRI gD4 aa 36 fwd	tatatatgaattcgttgttcgtggtaaccagaaccag
VK56	gD4 aa 280 rev ScaI	tatatatagtactttagtggtggtggtgatggtggccttggaagtacaggttttc aggaacaggacgagcgaag
VK61	gD1 D261N fwd	aggagagcatatgacatggttgaagttctggttcgtctacaatggtggaaaccta ccagtgcaaggatgacgacgataagtag
VK62	gD1 D261N rev	atgcctgggcttcataaaactgcactggtaggtttccaccattgtagacgaacca gaacttcacaaccaattaaccaattctg
VK63	gD1 F213A fwd	cttttctgtaactattcccagtgaacggtgtccgattgccgctgagcaaaacttt ggcaatccaggatgacgacgataagtag
VK64	gD1 F213A rev	ctggagttttacaccgatccggattgccaaagttttgctcagcggcaatcggaca ccgttcaccaaccaattaaccaattctg
VK65	gD4 D261N fwd	aggtgtacatttagcatgggtaaaacactggtttgtgcaaaatggtggaaacctt ccagtacaaggatgacgacgataagtag
VK66	gD4 D261N rev	acgcctgggcttcgtaaaactgtactggaaggtttccaccattttgcacaaacca gtgttttacaaccaattaaccaattctg
VK67	gD4 F213A fwd	cttttccgtaacaattccgagcagccattgtccgctttctgctgagcagaacttt ggtaatccaggatgacgacgataagtag
VK68	gD4 F213A rev	caggagttttacagcgatcaggattaccaaagttctgctcagcagaaagcggaca atggctgccaaccaattaaccaattctg
VK69	gD1 rever261 fwd	aggagagcatatgacatggttgaagttctggttcgtctacgatggtggaaaccta ccagtgcaaggatgacgacgataagtag
VK70	gD1 rever261 rev	atgcctgggcttcataaaactgcactggtaggtttccaccatcgtagacgaacca gaacttcacaaccaattaaccaattctg
VK71	gD1 rever213 fwd	cttttctgtaactattcccagtgaacggtgtccgattgcctttgagcaaaacttt ggcaatccaggatgacgacgataagtag
VK72	gD1 revr213 rev	ctggagttttacaccgatccggattgccaaagttttgctcaaaggcaatcggaca ccgttcaccaaccaattaaccaattctg
VK73	gD4 revert261 fwd	aggtgtacatttagcatgggtaaaacactggtttgtgcaagatggtggaaacctt ccagtacaaggatgacgacgataagtag
VK74	gD4 revert261 rev	acgcctgggcttcgtaaaactgtactggaaggtttccaccatcttgcacaaacca gtgttttacaaccaattaaccaattctg
VK75	gD4 revert213 fwd	cttttccgtaacaattccgagcagccattgtccgctttcttttgagcagaacttt ggtaatccaggatgacgacgataagtag

VK76	gD4 revert213 rev	caggagttttacagcgatcaggattaccaaagttctgctcaaaagaaagcggaca atggctgccaaccaattaaccaattctg
WA2	gD rev	acatgctcatatgttctccg

Table S 3: **Frequencies of contacts between MHC-I and EHV-1/4 gD residues and their assumed functions in Protein-Protein Interface of final peptide-containing docking pose.**

**MHC-I residue**	**EHV-1 gD**		**EHV-4 gD**		**Assumed function for Protein-Protein Interface**
	**Contacting residue**	**Frequency over MD trajectory [%]**	**Contacting residue**	**Frequency over MD trajectory [%]**	
R103	E242	100 ± 0	E241	0 ± 0	Additional contact sealing the binding interface
Y108	A157	100 ± 0*	N156	92 ± 11	O-ring for I166-F213/212 contact
N110	R222	92 ± 5	R221	41 ± 28	O-ring for I166-F213/212 contact
E113	R238	98 ± 3	R237	28 ± 37	Additional contact sealing the binding interface
**I166**	**F213**	**97 ± 4**	**F212**	**96 ± 4**	**Hot spot residue**
**R169**	**D261**	**100 ± 0**	**D260**	**99 ± 1**	**Hot spot residue**
A173	W257	97 ± 1	W256	86 ± 14	O-ring for R169-D261/260 contact

* The contact MHC-I Y108 – EHV-1 gD A157 summarizes lipophilic contacts between side chains and hydrogen bond between OH-group of Y108 and A157 backbone.
